# G-Tric: generating three-way synthetic datasets with triclustering solutions

**DOI:** 10.1186/s12859-020-03925-4

**Published:** 2021-01-07

**Authors:** João Lobo, Rui Henriques, Sara C. Madeira

**Affiliations:** 1 LASIGE, Faculdade de Ciências, Universidade de Lisboa, Campo Grande 016, 1749-016 Lisbon, Portugal; 2 INESC-ID and Instituto Superior Técnico, Universidade de Lisboa, Av. Rovisco Pais 1, 1900-001 Lisbon, Portugal

**Keywords:** Three-way data analysis, Three-dimensional data, Triclustering, Synthetic data generation, Unsupervised learning, Subspace clustering

## Abstract

**Background:**

Three-way data started to gain popularity due to their increasing capacity to describe inherently multivariate and temporal events, such as biological responses, social interactions along time, urban dynamics, or complex geophysical phenomena. Triclustering, subspace clustering of three-way data, enables the discovery of patterns corresponding to data subspaces (triclusters) with values correlated across the three dimensions (observations $\times$ features $\times$ contexts). With increasing number of algorithms being proposed, effectively comparing them with state-of-the-art algorithms is paramount. These comparisons are usually performed using real data, without a known ground-truth, thus limiting the assessments. In this context, we propose a synthetic data generator, G-Tric, allowing the creation of synthetic datasets with configurable properties and the possibility to plant triclusters. The generator is prepared to create datasets resembling real 3-way data from biomedical and social data domains, with the additional advantage of further providing the ground truth (triclustering solution) as output.

**Results:**

G-Tric can replicate real-world datasets and create new ones that match researchers needs across several properties, including data type (numeric or symbolic), dimensions, and background distribution. Users can tune the patterns and structure that characterize the planted triclusters (subspaces) and how they interact (overlapping). Data quality can also be controlled, by defining the amount of missing, noise or errors. Furthermore, a benchmark of datasets resembling real data is made available, together with the corresponding triclustering solutions (planted triclusters) and generating parameters.

**Conclusions:**

Triclustering evaluation using G-Tric provides the possibility to combine both intrinsic and extrinsic metrics to compare solutions that produce more reliable analyses. A set of predefined datasets, mimicking widely used three-way data and exploring crucial properties was generated and made available, highlighting G-Tric’s potential to advance triclustering state-of-the-art by easing the process of evaluating the quality of new triclustering approaches.

## Background

Current developments in technologies related to data collection, storing, and processing allowed several fields of expertise to start exploring new ways of describing their activities using the data they produce. Data that will, then, be analyzed to extract meaningful information to help decision-making processes or to draw conclusions about cases in study. In recent years, three-way data (observations $\times$ features $\times$ contexts), also referred as three-dimensional, tridiac, tensor, or cubic data, gained popularity due to their capacity to describe events related across several dimensions (three, in this case) and have properties that evolve with them. Different applications can be found across several domains, such as biological, medical, social, financial or geophysical data analysis [1].

In biology, three-way gene expression data, represented as *gene-sample-time* [2–4], is used to study how genes are expressed during the progression of a disease, or treatment, and unravel complex biological and physical processes that influence their evolution.

Three-way data are also capable of capturing behaviors and trends common to several individuals, being able to represent how communities function and respond together. Notable examples can be found in medical data analysis, where temporal patient data (patient-feature-time) [5] is used to describe patient profiles and disease progression patterns during patient follow-up. Alternatively, in social data [6], individuals’ preferences (*individual-feature-time*) and interactions (*individual-individual-time*) are collected to improve the contents provided, recommendations, to serve communities of users sharing similar tastes.

In the financial domain, these data are used to study trading and stock investing to improve profits. *Stock-ratio-data* [7] are used to relate stock prices and their respective financial ratios during a time interval and can be used to identify groups of stocks whose performance on different indicators can influence their prices.

To foster knowledge discovery from three-way data, further advances are needed in *triclustering* [1], a new subspace clustering technique, proposed to enable the search for patterns that correlate subsets of observations, showing similarities on a specific subset of features, and whose values are repeated or evolve coherently across a third dimension, generally time or space.

Several *triclustering* algorithms were already proposed [1], based on different approaches, able to find different types of patterns, with distinct structures and tolerating noise and/or missing values. These approaches range from heuristic-based methods to exhaustive ones, to balance the complexity of the task (NP-Hard [1]) with the number of patterns that they can find. In this context, a key task during the development of a new algorithm is the evaluation of how good the found solutions are, where a triclustering solutions is a set of triclusters. This evaluation is usually performed by testing the new method with available data and checking the quality of the found triclusters using a predefined set of metrics evaluating different properties, such as homogeneity, size or statistical significance.

Real datasets are used in general during these tests, but this procedure has significant limitations. Since there is no previous knowledge about the type of patterns that are expected to be found, there is no ground truth, that is, a known baseline solution that can be compared with the algorithm’s output to assess its effectiveness, besides its efficiency. This means that each new algorithm can find different groups of triclusters, outputting a triclustering solution with distinct size and characteristics. This makes it is difficult to establish an objective and independent criteria to evaluate them.

Synthetic datasets are one way to surpass this limitation. These data can be customized, generated containing specific properties, defined by the author, and a set of planted triclusters (triclustering solution) with known structures, and then used to better assess algorithms’ performance using ground truth.

Despite the inherent advantages of generating triclustering data, to our knowledge, no three-way data generator is available to allow the generation of triclustering solutions. Therefore, each author has to generate their own data. This task is critical, can be time consuming, and even assuming synthetic data is generated correctly their properties can be, and usually are, biased towards the triclustering algorithm under evaluation. Furthermore, they are then used to compare the new algorithm with the state-of-the-art, in turn proposed and evaluated using other data, compromising the validity of some comparisons, and making them unfair, even if experimentally correct. Several authors [2, 3, 5, 6] generated specific data to test their algorithms.

In this context, we propose a new synthetic data generator, *G-Tric*, able to generate three-way datasets with planted triclusters (triclustering solution), where the user can define several properties regarding the dataset and the planted solutions (customized dataset and solution properties). Concerning dataset properties, the generator can create numeric or symbolic data, with default or custom alphabets, using backgrounds following predefined statistical distributions, and allowing a predefined amount of noise, missing values, and errors. Regarding solution properties (the planted triclusters), the user can define: (1) how many triclusters should be planted (solution size) and how their structure is defined using statistical distributions; (2) what type of patterns should to be planted; (3) what are the overlapping properties of triclusters; and (4) what is the amount of noise, missing values, and errors allowed in each tricluster. We also ensure the user to be able to generate datasets with varying sizes without worrying with scalability issues.

Besides the ability to easily generate customized three-way data with triclustering solutions, the proposed generator enables the possibility to perform benchmarks on existing algorithms to study their efficiency within certain conditions, or their effectiveness in finding different types of patterns, by allowing the creation of several datasets with an extensive board of characteristics. This provides the unprecedented opportunity to comprehensively assess the strengths and limitations of state-of-the art and new triclustering algorithms, promoting the advance in the area of three-way data analysis. To this end, we provide an initial set of generated benchmark datasets, that can then be extended using the software.

The paper is organized as follows. The rest of this section defines the triclustering task and its associated properties, such as coherence, quality, and evaluation methods. Section "Related work" reviews the *state-of-the-art*, concerning synthetic data generation. Section "Implementation" briefly discusses the software architecture, presents a possible representation for the problem, and describes and exemplifies each feature of the generator. Section "Results" presents the set of datasets generated, identifying the kind of problems they describe, and the associated properties. Finally, section "Conclusions" draws conclusions. As supplementary material (Additional file 1), we further provide a guide containing the mapping between the set of properties the user can define to create a new dataset and displaying the respective way of doing it in the interface. By using a toy example, this example can serve as a tutorial.

### Triclustering task

#### Definitions

##### **Definition 1**

A **three-way dataset**, also termed three-dimensional dataset, *D*, is characterized by *n* observations $X = \{x_1, \ldots , x_n\}$, *m* features $Y = \{y_1, \ldots , y_m\}$ and *p* contexts $Z = \{z_1, \ldots , z_p\}$. Analogous to 2D data-matrices, the data in 3D datasets can be real-valued or symbolic. Each element, $a_{ijk}$, relates an observation $x_i$, an attribute $y_j$ and a context $z_k$ [1].

**Fig. 1:**
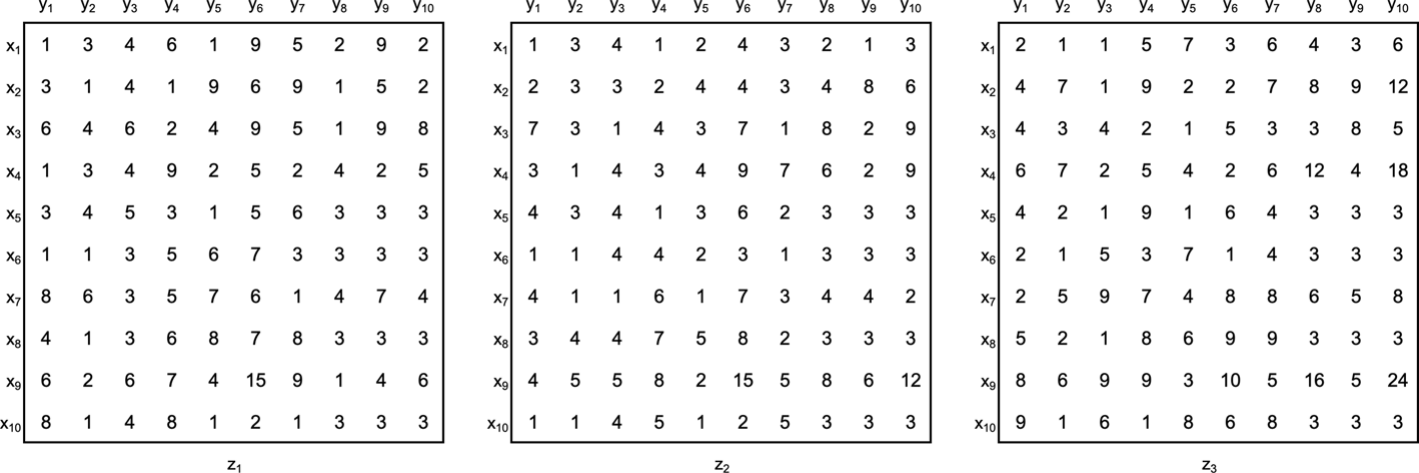
Example of Dataset D (10 observations $\times$ 10 features $\times$ 3 contexts). Each 2D matrix represents a context with 10 observations $\times$ 10 features

This kind of datasets allows the representation of temporal data, when contexts correspond to time points. If the value of a particular object is fixed, such as observation, a features, or a context, a 2D data matrix is obtained, being called a **slice**. Figure 1 shows an illustrative dataset *D* represented as a set of slices according to the size of the context dimension.

##### **Definition 2**

Given the 3D dataset *D*, a **tricluster**, $T = (I,J,K)$, is a subspace of the original dataset, where $I \subseteq X$, $J \subseteq Y$ and $K \subseteq Z$ are subsets of observations, features and contexts, respectively [1].

##### **Definition 3**

**Fig. 2:**
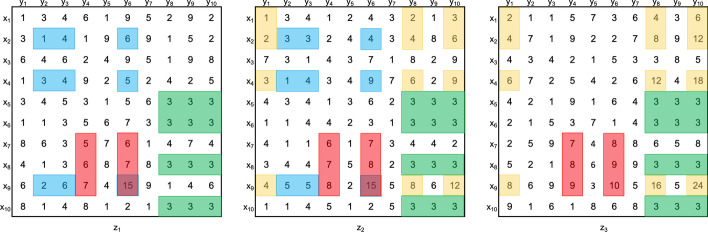
Dataset *D* highlighting a triclustering solution with four triclusters, colored blue, red, green and yellow

**Fig. 3:**
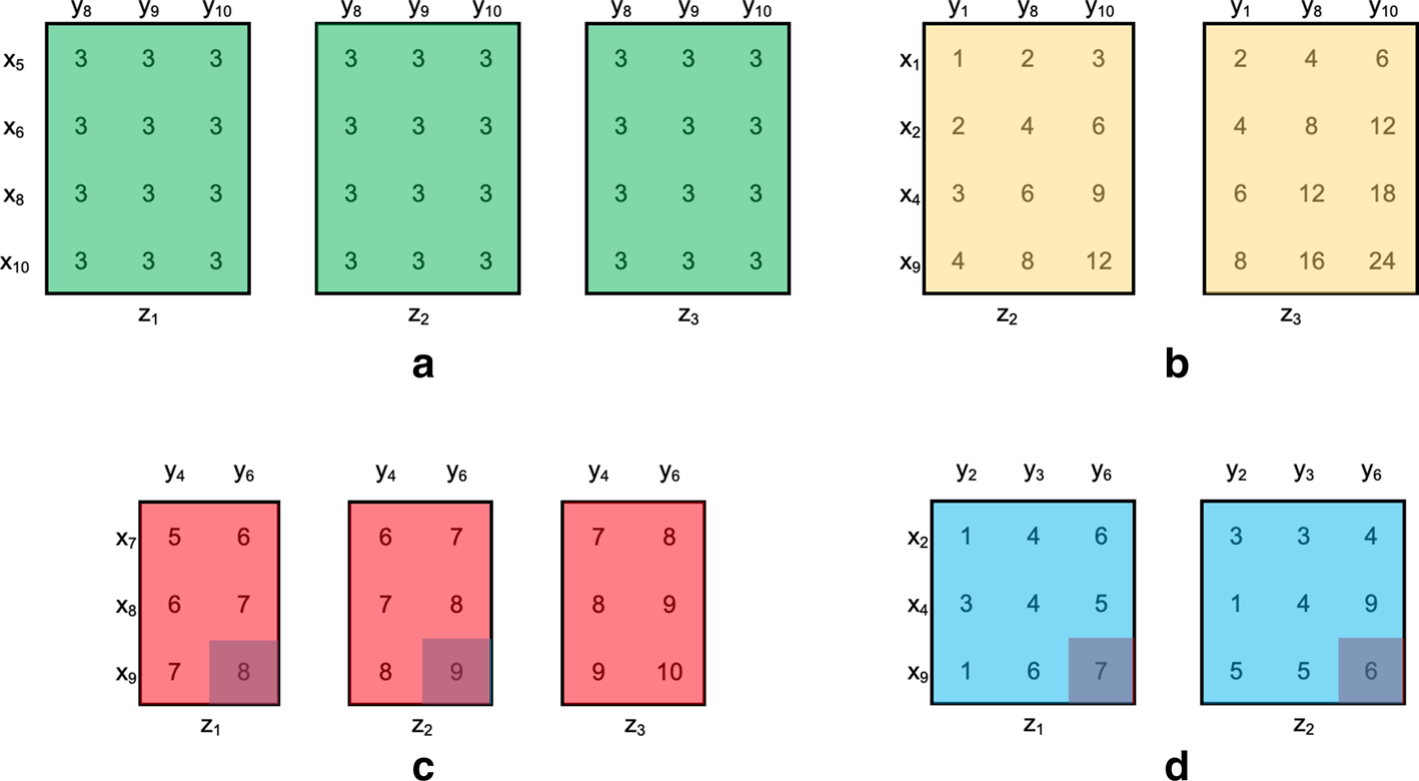
Set of triclusters found by a triclustering task on dataset D in Fig. 1 and highlighted in Fig. 2 (triclustering solution of size 4). Since there is an overlapping between triclusters **c** and **d**, the elements $(x_{9},y_{6},z_{1})$ and $(x_{9},y_{6},z_{2})$ represent the individual contributions for the additive plaid model presented in Fig. 2

In this context, the **triclustering task** consists in finding the set of triclusters $T = \{t_1, \ldots , t_n\}$, such that each $T_i \in T$ satisfies specific properties, such as, homogeneity and statistical significance, as defined below [1]. Figure 2 shows the dataset with the set of triclusters resulting from a triclustering task (a triclustering solution) highlighted. Figure 3 shows, in detail, the four triclusters in these triclustering solution.

#### Coherence

The types of patterns that the triclustering task is able to find are defined by the type of coherency that the desired subspaces can express. These subspaces can be correlated according to the following assumptions:

**(1) Constant**: subspaces that exhibit constant [symbolic data (Eq. 1)] or approximately constant [real-valued data (Eq. 2)] values.

1 $$\begin{aligned} a_{ijk}&= c, \end{aligned}$$

2 $$\begin{aligned} a_{ijk}&= c + \eta _{ijk}, \end{aligned}$$

where $a_{ijk}$ is the value of observation *i*, feature *j* and context *k*, *c* is the common value (seed) and $\eta _{ijk}$ corresponds to noise. Figure 3a shows an example of a constant tricluster.

**(2) Additive**: where each element is correlated through the sum of a factor from each dimension,

3 $$\begin{aligned} a_{ijk} = c + \alpha _i + \beta _j + \gamma _k + \eta _{ijk}, \end{aligned}$$

where $\alpha _i$, $\beta _j$ and $\gamma _k$ are contributions from observation $x_i$, feature $y_j$ and context $z_k$. The assumption can be fully additive when $\alpha _i \ne 0$, $\beta _j \ne 0$ and $\gamma _k \ne 0$, or partially additive otherwise [1]. Figure 3c shows and example of a full additive triclusters with $c = 2$, $\alpha _i = \gamma _k = \{1,2,3\}$, and $\beta _j = \{1,2\}$.

**(3) Multiplicative**: when the tricluster can be obtained through a product of terms from each dimension,

4 $$\begin{aligned} a_{ijk} = c * \alpha _i * \beta _j * \gamma _k + \eta _{ijk}, \end{aligned}$$

where $\alpha _i$, $\beta _j$ and $\gamma _k$ are contributions from observation $x_i$, attribute $y_j$ and context $z_k$. The assumption can also be full or partial multiplicative. Figure 3b shows an example of a full multiplicative tricluster, where $c = 1$ and $\alpha _i = \beta _j = \{1,2,3\}$, and $\gamma _k = \{1,2\}$.

**(4) Order preserving**: when instead of looking at the actual tricluster’s values, the search goal is to find linear orderings across a specific dimension. For example, a matrix can be order preserving across columns, if there is a permutation of the said dimension for which each row has an increasing sequence of values [8]. On a three-dimensional dataset, it is expected that this ordering is maintained across different contexts. An order preserving tricluster across columns is shown in Fig. 3d.

#### Plaid effects

Unlike other clustering methods, and similarly to biclustering [9, 10], in triclustering an element (observation, feature or context) can be part of more than one cluster (tricluster), or in none. When a set of elements belong, simultaneously, to a group of triclusters, we are in the presence of **overlapping triclusters**. Overlapping regions between two or more triclusters can be described in accordance to a *plaid* assumption. Under this assumption, the value of an element that participates in multiple triclusters is a function of the expected value from each tricluster. As such, elements are defined using the cumulative effects of the overlapping triclusters considering two assumptions: *additive* (5) or *multiplicative* (6), where each element is computed by the sum or the product of the individual contributions of each tricluster.

5 $$\begin{aligned} a_{ijk}= {} c + \sum _{t = 0}^{q} \theta _{ijkt} \rho _{it} \kappa _{jt} \tau _{kt}, \end{aligned}$$

6 $$\begin{aligned} a_{ijk}= {} c + \prod _{t = 0}^{q} \theta _{ijkt} \rho _{it} \kappa _{jt} \tau _{kt}, \end{aligned}$$

where $\theta _{ijkt}$ defines the contribution from tricluster $B_t = (I_t, J_t, K_t)$ and $\rho _{it}$, $\kappa _{jt}$ and $\tau _{kt}$ are binary values that indicate if observation *i*, attribute *j* and context *k* are present on tricluster *t*. Figure 2 shows an overlapping example, with an additive plaid assumption, between the red and blue triclusters. Figure 3 reveals the individual contributions of each tricluster in the overlapped region.

#### Quality

The triclustering task should be able to tolerate predefined levels of *noise* (deviations from the expected values) within the data under study, as well as, *missing data* (values of a particular observation that are not available) or *errors* (values whose deviation level is higher than that found in noisy elements, caused by incorrect measurements, for example). The higher the amount of these defective values, the lower the quality of the data, impacting the desired coherency for a given subspace.

### Evaluation

After obtaining a triclustering solution, it is necessary to evaluate its correctness and quality, to be able to compare solutions. This analysis should be carried out using metrics that measure different views, with two existing goals: *single solution evaluation* and *comparison between solutions*.

#### Single solution evaluation

The first perspective tries to assess how good a found solution is by evaluating its quality across different performance views. This solution can be evaluated either by **extrinsic** methods, where a known ground truth exists, or by **intrinsic** methods, where there is not any prior information about the subspaces that can be present on the dataset under study.

Regarding **extrinsic** metrics, generally, a set of known triclusters is planted on the dataset, and each algorithm is supposed to find them. The solutions found are then compared with the planted subspaces, and the higher the number of shared elements is, the better they are. This comparison can be made using metrics based on *F-Measure* [11] and *Jaccard-based scores* [6], for example, or the *3D Revised Match Score (RMS3)* proposed by Henriques and Madeira [1].

The solution’s **intrinsic** quality can be computed by evaluating its coherence, by calculating its degree of intra and interplane homogeneity, that is, correlation between values across two or more dimensions, using metrics such as *MSR* [12], *Pearson* [2] and *Spearman* correlations [13], that can be extended to a third dimension, or *Mutual Information Score* [14].

In addition, metrics that consider the statistical significance of a tricluster can be beneficial to distinguish true triclusters from random patterns on the dataset. This would allow a reduced number of false-positive (triclusters that appear by chance) and the number of false-negative (real triclusters that are excluded from the solution) [1]. This evaluation can be done through methods that analyze deviations between the observed data and the underlying data distributions [15], thresholding methods [16], or size expectations collected from randomized data [17]. However, these tests are limited by the allowed homogeneity criteria and placed assumptions on the underlying data.

Another view on the quality of the solution is to evaluate how insightful and meaningful the triclusters found are to the problem at hand. How actionable are the triclustering results? For instance, in biological domains [3, 5, 13], *functional annotations* and *gene ontologies* are used to extract meaning from the sets of genes found and to understand why they are correlated.

#### Comparison between solutions

Comparison of triclustering algorithms is also key to identify their relative strengths and weaknesses. Relevant comparisons can be made either by studying the inherent structure and coherency of the produced solutions, establishing a framework of comparison, and combining it with information about the background or prior information of planted solutions. Also, by their actionability and relevance to the problem to which the algorithms are applied.

In this context, the extrinsic metrics, discussed above to evaluate one triclustering solutions, can be extended to compare solutions produced from different algorithms. The intrinsic scores achieved by different algorithms can also be compared side-by-side. According to Horta and Campello [18], in order to perform a correct analysis of the quality of each biclustering solution, the evaluation metrics should respect eight properties that are intended to favor algorithms than can clearly distinguish different patterns. In particular, the ability to retrieve maximal subspaces, that is, subspaces that are not included on a larger subspace, and can do so without adding noise to increase the subspace’s area. The authors studied 14 similarity measures and verified the ones that respect most of the properties were the *Clustering Error (CE)* [19] and a measure of soft-clustering, *CSI*. Since triclustering is an extension of biclustering, both metrics could be extended and used to evaluate triclustering solutions.

Authors proposing triclustering algorithm have also defined comparison metrics to test the performance of the developed algorithms against the existing state-of-the-art. Bhar et al. [20] used a set of metrics, such as *TQI*, *Affirmation Score*, *Coverage* and *SBD* to perform the comparisons. Gutiérrez-Avilés et al. [21] proposed a new metric, *TRIQ*, to evaluate triclustering algorithms by combining correlation measures, graphic validation, and functional annotations, combining this way the coherency expressed with the relevance of the found subspaces to the problem.

Triclustering algorithms should also be evaluated on their ability to tolerate noise. The *Adjusted Rand Index* [13] and *Jaccard Similarity Coefficient* [6] have been considered to this end. Furthermore, and given the complexity and often size of the three-way data to be analysed, efficiency and scalability should also be of great concern. Thus, the ability to handle different dataset sizes, the memory consumption and execution time needed constitute additional and important criteria to consider when deciding which algorithm is better.

### Related work

The generation of synthetic data is advantageous to test specific algorithm’s properties. Real data are, sometimes, difficult to obtain, and it is impossible to control the peculiarities they exhibit. In this context unsupervised learning tasks, including pattern mining and (subspace) clustering tasks, frequently resort to synthetic generators, as shown below, to produce custom data describing distinct problems to potentiate the work developed, facilitate analysis, and comparisons.

In pattern mining, Omari and Conrad [22] proposed a generator to create datasets consisting of transactions that record purchases, with an associated timestamp to study customer buying habits. Generators are also useful for *context-aware recommender systems*, as they allow to create sets of actions taken by users with some context information that describe them [23]. Machine learning techniques, such as image recognition, also benefit from these tools, with a generator based on *generative adversarial networks* that produces image-based datasets with demographic parity [24] being an example. Statistical learning methods can also be trained using data produced using the *Bayes* framework [25] also resorting to synthetic data. Other domains, such as software testing [26] or the development of anonymization techniques [27], also make use of synthetic data.

In clustering, some tools were also proposed to facilitate algorithm evaluation. One of them, proposed by Pei and Zaiane [28] enables the creation of datasets with planted clusters based on the user’s requirements, such as the number of points, the number of clusters, the size, shapes, and locations of clusters, and the density level of either cluster data or noise/outliers in a dataset. With the goal of performing clustering and outlier detection analysis. In biclustering, several benchmark contributions were made through the generation of synthetic data, with some of them providing the tools needed to replicate them. In this context, BiMax [29], produces datasets with biclusters with varying degrees of noise and overlapping, but is not capable of producing dynamic structures, with different sizes and coherencies. BiBench [30] was also proposed to fulfill some of the limitations of BiMax, by allowing the generation of datasets with different sizes and different numbers of biclusters with shift and scale patterns (additive and multiplicative). However, BiBench assumes only constant values across columns, preventing the generation of observations with different, yet correlated values. It does not consider order-preserving patterns, and the biclusters have fixed dimensions. BiGen was later proposed by Henriques [31] to correct these limitations by allowing the generation of biclusters of both symbolic and numeric natures, with varying sizes (each dimension is described through a statistical distribution), with more and different patterns, and with parameterizable overlapping and quality (noise and missings) settings. This is the data generator used to evaluate all the algorithms made available in BicPams software [32].

Concerns triclustering, several algorithms were developed and tested on synthetic data produced by the authors [1]. Unfortunately, none of them made available the respective generators. RSM and CubeMiner [33] were evaluated using IBM’s Quest Data Generator,^1^ even though this generator is more suitable for pattern mining datasets since it generates sets of transactions. Three-way data generators are scarce, and, to the best of our knowledge, there is no generator producing three-way data with planted triclusters to be used to foster the research on triclustering algorithms and three-way data analysis. For that reason, G-Tric used BiGen [31] as a basis to develop a generator able to create 3D datasets with planted triclusters, interacting with each other and having varying properties. Besides the introduction of a third dimension, G-Tric also adds new features to BiGen, such as allowing the definition of a pattern to each dimension (in BiGen, a specific coherency was applied to one dimension while the other was filled with non-constant elements), dividing the quality parameters in two sets, one for the dataset’s background and the other for the subspaces planted (unlike the global definitions of BiGen). In G-Tric, the background elements can follow a discrete distribution, where each symbol/element follows a user-defined probability. Moreover, the overlapping settings were extended so that the user can choose how many triclusters can overlap and the number of elements they can share.

## Implementation

**Fig. 4:**
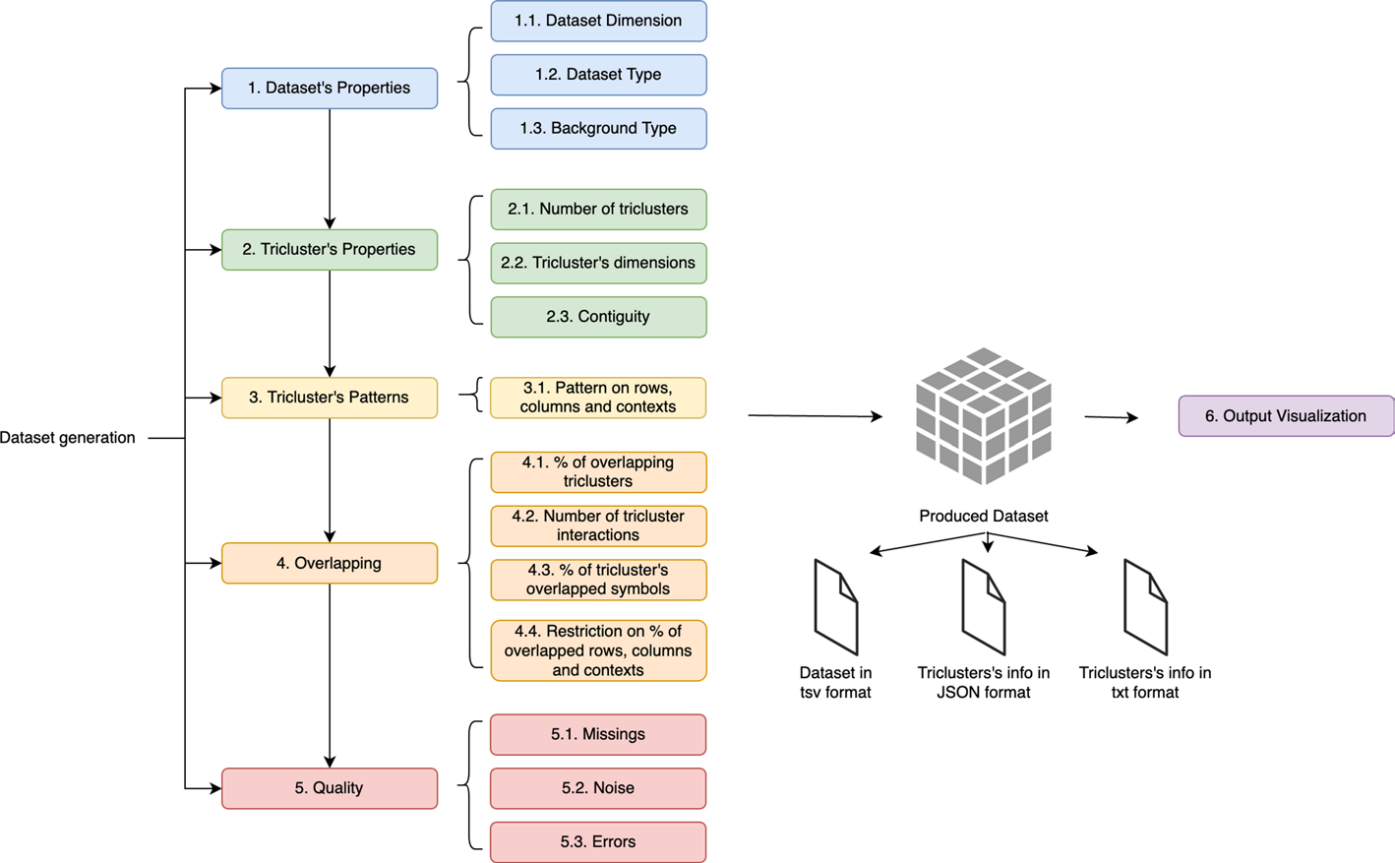
Generation stages

To create fully customized datasets, G-Tric requests the user to define desirable properties related to both the dataset and the triclusters, as described by the workflow of Fig. 4. For all of them, default values are automatically inserted and the user can choose whether to control the generation of the whole dataset properties or to explore only a subset of properties of interest. A dataset is defined by its type (symbolic or numeric), its dimensions, and the distribution of values in its background (data in the absence of local correlations).

Regarding the triclusters, the user can define how many will be planted and how is their structure generated, using statistical distributions to choose how many rows, columns, and contexts each one will have. The other tricluster-related properties to be defined are the set of patterns to use while composing the planted solution and the level of overlap between triclusters.

The last customization step is related to the quality of the data and is where the amount of missing values, noise, and errors to plant are defined. For each of these quality criteria, the user can decide the percentage of affected elements for the triclusters and the background, separately.

The number and characteristics of the generated three-way dataset and planted triclustering solutions that the user can control resemble the properties commonly found in 3D real data.

After generating the new dataset, a new feature is made available in the interface so that the user can visualize graphically the set of triclusters planted, which are the patterns chosen, where they are located, and how they evolve across the context dimension.

The following subsections will detail the properties and parameter set made available by the generator. The dataset *D*, introduced earlier (Figs. 1, 2), will be used as a toy example to demonstrate how the generator can simulate real-world datasets.

### G-Tric overview

Before starting the development of the proposed generator, the first step was to define the best way to represent a tricluster, and its associated properties, in an efficient and low memory consumption way.

Since a tricluster can be seen as a set of correlated biclusters, the chosen model represents a tricluster as a single bicluster (template) that is repeated through a set of slices, according to the third dimension. This template is supposed to have three main characteristics: (1) seed (integer or real-valued for numeric triclusters, or a 2D matrix for symbolic ones), (2) row and column patterns, and (3) set of row and columns factors (only for numeric triclusters). The definition of symbolic triclusters is slightly different due to its non-numeric nature.

**Fig. 5:**
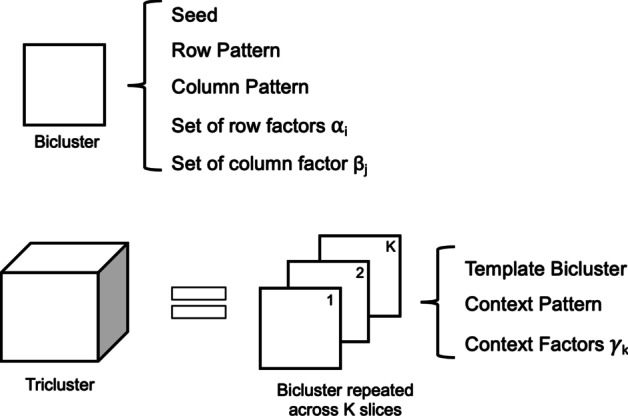
How a numeric tricluster is represented in the software

**Fig. 6:**
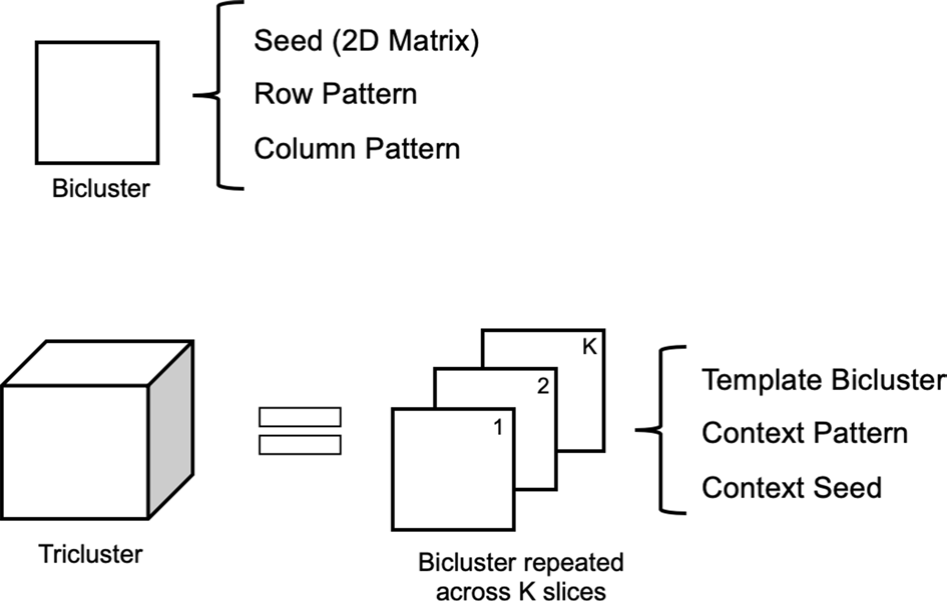
How a symbolic tricluster is represented in the software

A single tricluster contains one template, and the information needed to repeat this template across the context dimension, together with the context pattern. In case of a numeric tricluster, the information refers to the context factors that enable the use of Eqs. (3) and (4) to represent the tricluster’s coherency, as illustrated in Fig. 5. In case of a symbolic tricluster, the tricluster is represented either by the repetition of the template seed across the context dimension (in the case where a constant pattern across contexts exist) or by a particular seed for each context, illustrated by the ’context seed’ in Fig. 6 (in the case where the tricluster is only coherent across the row or column dimensions).

Following this model, there is no need to store all the values of a tricluster, since they can be obtained by correlating the respective seed with the dimension’s factor, as shown by Eqs. (1) to (4). The template bicluster also stores the set of rows and columns where it is located, while the tricluster stores the context identifiers, where the template is present and the plaid coherency assumed.

This structure is reflected on the generator through the *Bicluster*, *Tricluster* and *Slice* classes.

Given that each dataset/tricluster set can be numeric or symbolic, the software has to handle different data types and subtle structural differences between these two types of datasets/triclusters. This was done through the use of the inheritance concept, using a set of abstract classes such as *Generator*, that is responsible for creating the dataset and plant the triclusters; *Dataset*, that is composed of the background and a set of triclusters; and *Bicluster*, and *Tricluster*, that are based on the structure presented in Figs. 5 and 6.

**Fig. 7:**
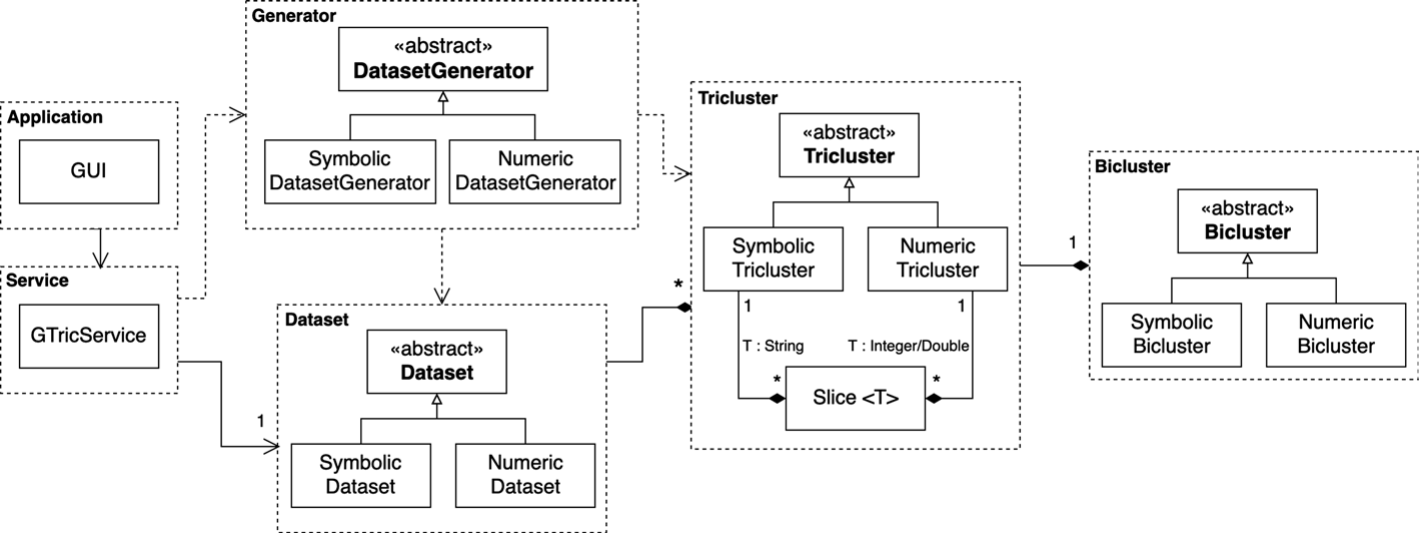
Simplified domain model showing the interactions between the main classes involved on the generation process

The *GUI* (Graphical User Interface) was developed using JavaFX, and uses a dedicated class, *GTricService* to allow communication between the interface and the business layer. Figure 7 exemplifies the described organization using a simplified domain model, containing the main classes of the generator. The visualization of the output was performed using an open-source library for Java, called *XChart*,^2^ modified to allow the creation of Heat Maps with missing and non-numeric (symbolic dataset) values. An additional file is made available, as supplementaty material, to provide guidelines for using G-Tric to generate a particular dataset, providing a mapping between the set of properties describing the dataset (type, structure, patterns, overlapping and quality, as described below) and GUI parameters.

### Symbolic and numeric datasets

The first step to generate a new dataset is to define its type and dimensions. To this end, the user has to define the following parameters related to the dataset’s structure only:

1. **Dataset type**: Regarding the dataset type, G-Tric allows the creation of both *symbolic* and *numeric* datasets. This choice will influence the kind of patterns that can be planted (more on section "Tricluster patterns"). The *symbolic* type is useful when the experiment that the user wants to reproduce is better represented by symbols, and the trends shown by data are more interesting than the actual values that cause them. For other studies, a *numeric* dataset can be used, with either *integer* or *real-valued* elements;
2. **Dimension**: The dimension ($X \times Y \times Z$) parameter defines the actual structure of the dataset, as it is requested to the user to indicate how many observations, |*X*|, features, |*Y*|, and contexts, |*Z*|, are going to be generated;
3. **Alphabet**: The set of symbols, or the range of values in the dataset. For *symbolic* datasets, the user can define the length, *n*, of the alphabet, and the generator will create a default set of *n* symbols. Alternatively, the user can indicate a list of preferred symbols and G-Tric will use it. In this case, the order in which the symbols are provided determines the ordering of the alphabet. *Numeric* datasets are limited by a minimum and maximum value;
4. **Background distribution**: The last step regarding the datasets structure is the definition of how the alphabet will be distributed across the 3D matrix, that is, the background. To help with this, G-Tric makes available four types of backgrounds to choose from: *(1) Uniform*: assuming each alphabet element has the same probability to fill a certain position of the matrix; *(2) Normal*: that uses the *Gaussian* distribution to generate the background; *(3) Discrete*: allowing the user to define the probability associated to each symbol (this type of background is only available on *symbolic*, or *numeric* datasets with integer values); and *(4) Missing*: assuming a null background, i.e. elements that do not belong to a pattern are missing.

### Tricluster structure

After deciding the properties of the dataset, the next task is to define the characteristics of the triclusters to be planted, starting by the amount and structure they will express:

1. **Number of triclusters to plant:** The first parameter allows the user to define the number of triclusters that are going to be planted. The size of the planted subspaces will limit this number. In the current version, if the user inserts a large number of triclusters, possibility preventing the software to plant all of them, G-Tric will try to maximize the number of placed triclusters and will output this;
2. **Dimension:** To define the dimension of the triclusters, G-Tric, again, makes available two distributions, *Normal* and *Uniform*, for each dimension, not only to select how many *observations*, *features*, and *contexts* each tricluster will have, but also to define how their size will vary. Allowing the creation of flexible structures;
3. **Contiguity:** The last parameter regarding the triclusters properties is the contiguity that is useful to simulate certain types of data, such as time-series. G-Tric allows the existence of triclusters with a contiguous dimension, that can be either the *features* or the *contexts*.

The positioning of triclusters in a given three-dimensional dataset follows a set of principles that starts with a random selection of a subset of objects from each dimension, $I \subseteq X, J \subseteq Y, K \subseteq Z$, to avoid biases associated with random point selection followed by side-specific extension of the subspaces. The settings related to size selection, using Uniform and non-Uniform distributions, and overlapping properties allow the user to further define regions with higher coherence density on a specific dimension. For datasets that are temporal in nature, the selection of objects along the time dimension can be contiguous, following the Uniform distribution for selecting the starting time point, $U(0, T - |K|)$, or non-contiguous.

### Tricluster patterns

The crucial aspect regarding triclusters is the type of coherency they show. This is what makes them attractive relative to the background data and is what guides every algorithm. Triclustering patterns are the available way to express the existing coherency across the cluster’s values.

As explained in section "Coherence", there are four types of coherency that a tricluster can have: *order preserving*, *constant*, *additive*, and *multiplicative*. G-Tric makes available four kinds of patterns, each one corresponding to the shown type of coherency.

Each tricluster’s dimension has a corresponding pattern, and all of them can have any one of the four available above or one more called *None*. This new pattern is useful to represent dimensions that do not have any coherency, considering that to have a triclustering is it only needed coherency over a single dimension. In short, the five patterns G-Tric makes available are: *Order Preserving*, *Constant*, *Additive*, *Multiplicative*, and *None*.

To represent a tricluster, a triple of patterns, $(P_{row}, P_{col}, P_{ctx})$, is used and each element represents the specific pattern applied to each dimension, determining the value for the respective term in Eqs. (3)–(4), or the respective template/context seed. For example, in a numeric tricluster, for an *Additive* (or *Multiplicative*) tricluster, Eq. (3) [or (4)] is used and the *Additive* (or *Multiplicative)* pattern in any dimension represents the existence of a particular factor and a *Constant* pattern sets the factor to 0 (or 1). In a symbolic tricluster, the dimension where the *None* pattern is applied determines the existence of a single seed (template seed), when it is applied to either the observation and feature dimension, or the need for a set of context seeds, when it is applied to the context dimension.

Even though a single dimension can have any pattern, there are some restrictions on the allowed set of patterns that can be combined to form a triple. For example:

- The *Order Preserving* pattern can only be attributed to a single dimension, and the other two must not show any type of coherency, that is, must have the *None* pattern;
- The *Constant* pattern can only be combined with either itself, the *Additive* or the *Multiplicative* one;
- *Additive* and *Multiplicative* patterns cannot be combined. This restriction exists because of the way a tricluster’s additive/multiplicative model is defined [see Eqs. (3) and (4)].
- The *None* pattern can only be used with *Order Preserving* and *Constant* triclusters.

Moreover, the type of dataset influences the allowed set of tricluster patterns that can be used. Since *symbolic* datasets can be formed using non-numeric symbols, it is not possible to use the *Additive* or *Multiplicative* patterns. On the other end, *numeric* datasets have no restrictions.

**Fig. 8:**
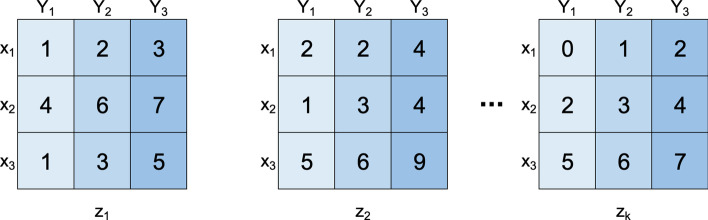
Tricluster with *Order Preserving* pattern on columns

The set of available pattern triples, and information about which ones can be used in both types of datasets are summarized in Table 1. Figure 8 shows and example of how an *Order Preserving* pattern on columns looks like. When the *Order Preserving* pattern is applied on the context dimension, to be able to simulate time profiles that resemble real-world data, such as gene expression time series data [34], G-Tric makes available three types of temporal patterns (*Monotonically Increasing*, *Monotonically Decreasing* and *Random*), to replicate processes that exhibit monotonic increasing or decreasing functions, or none.

**Fig. 9:**
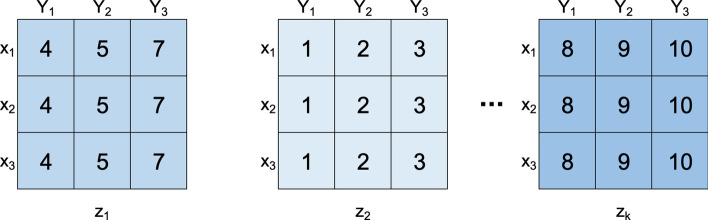
Tricluster with *Constant* rows but with an *Additive* pattern across columns and contexts, where, $c = 1$, $\alpha _i = \{0,0,0\}$, $\beta _j = \{1,2,3\}$, $\gamma _k = \{2,-1,6\}$, and $\eta _{ijk} = 0$

**Table 1:** Available patterns of each dataset’s type

Rows	Columns	Contexts	Symbolic	Numeric
Order preserving				
Order preserving	None	None	✓	✓
None	Order preserving	None	✓	✓
None	None	Order preserving	✓	✓
Constant				
Constant	Constant	Constant	✓	✓
None	Constant	Constant	✓	✓
Constant	Constant	None	✓	✓
Constant	None	Constant	✓	✓
Constant	None	None	✓	✓
None	Constant	None	✓	✓
None	None	Constant	✓	✓
Additive				
Additive	Additive	Additive	✗	✓
Additive	Additive	Constant	✗	✓
Constant	Additive	Additive	✗	✓
Additive	Constant	Additive	✗	✓
Additive	Constant	Constant	✗	✓
Constant	Additive	Constant	✗	✓
Constant	Constant	Additive	✗	✓
Multiplicative				
Multiplicative	Multiplicative	Multiplicative	✗	✓
Multiplicative	Multiplicative	Constant	✗	✓
Constant	Multiplicative	Multiplicative	✗	✓
Multiplicative	Constant	Multiplicative	✗	✓
Multiplicative	Constant	Constant	✗	✓
Constant	Multiplicative	Constant	✗	✓
Constant	Constant	Multiplicative	✗	✓

Figure 9 instantiates a tricluster, *T*, of size $|I|\times |J|\times |K| = 3\times 3\times 3$, that follows an additive model. In this case, the triple used is (*Constant*, *Additive*, *Additive*), and is mapped to Eq. (3), where the seed is $c = 1$, set of row’s contributions is $\alpha _{i} = 0$, the set of column’s contributions is $\beta _{j} = \{1,2,3\}$, the factors for the context dimension are $\gamma _{k} = \{2,-1,6\}$, and where there is no noise associated, $\eta _{ijk} = 0$. This example illustrates how a group of similar observations evolve together along the contexts. Multiplicative patterns work analogously but follow the multiplicative model in Eq. (4).

### Overlapping properties

**Fig. 10:**
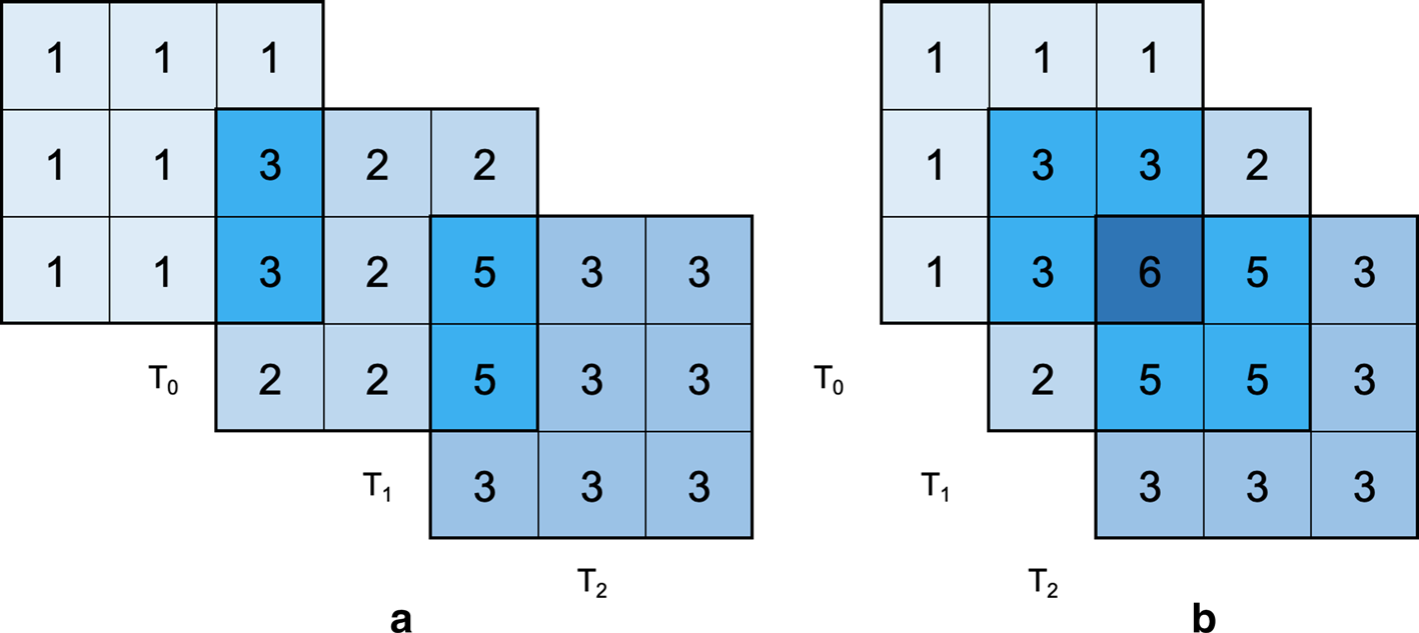
Two possible ways of overlapping, with additive plaid coherency, between a set of slices of three triclusters (for simplicity sake we are omitting the representation of the slices along a third dimension)

One relevant characteristic of triclustering is the possibility of several triclusters to overlap each other, sharing their observations and attributes on certain contexts. In G-Tric, the user can control the amount of overlapping allowed, as well as the type of coherency assumed on the overlapped region:

1. **Plaid coherency:** *Plaid coherency* defines how the values shared by a group of *n* triclusters are correlated. This coherency can follow four possible assumptions: *(1) Additive*, as defined in Eq. (5), where each value is composed by the sum of the *n* individual values of each tricluster; *(2) Multiplicative*, as in Eq. (6), that does similarly to the *Additive* using the product; *(3) Interpoled*, introduced by BiGen, where each value is obtained by calculating the average value of the corresponding tricluster’s elements, as described in Eq. (7),
  7 $$\begin{aligned} a_{ijk} = c + \frac{\sum _{t = 0}^{q} \theta _{ijkt} \rho _{it} \kappa _{jt} \tau _{kt}}{q}, \end{aligned}$$
  where $\theta _{ijkt}$ defines the contribution from tricluster $B_t = (I_t, J_t, K_t)$ and $\rho _{it}$, $\kappa _{jt}$ and $\tau _{kt}$ are binary values that indicate if observation *i*, attribute *j* and context *k* are present on tricluster *t*; and
  *(4) None*, where the value on the dataset will correspond to the respective element of the last tricluster generated;
2. **Percentage of overlapping triclusters:** Defines how many of the dataset’s triclusters can overlap. If the dataset has $|T| = 10$ triclusters, and $O_{triclusters} = 50\%$, then only half of the dataset’s triclusters will overlap;
3. **Maximum number of overlapping triclusters simultaneously:** The user can also control how many triclusters overlap together. This parameter, *k*, separates the set of overlapping triclusters in |*T*|/*k* groups. Furthermore, at each group, a set of intersections is generated between triclusters and can range from only pair-wise interactions, where triclusters overlap two by two, to a maximum of *k* wise interactions, where all triclusters share something. For example, in a dataset with $|T| = 10$, $O_{triclusters} = 100\%$, and the maximum number of triclusters that can overlap together is $k = 3$, we will have $10/3 \approx 3$ groups of overlapping triclusters, and a last group, with only one, that will not overlap with any other. In the overlapping groups, each one will have 3 triclusters, so, we can have interactions from something like $T_0 \cap T_1 \ne \emptyset , T_1 \cap T_2 \ne \emptyset , T_0 \cap T_2 = \emptyset$, as in Fig 10a, to $T_0 \cap T_1 \cap T_2 \ne \emptyset$, represented in Fig. 10b;
4. **Maximum percentage of overlapping elements:** lets the user define the maximum number of elements of a tricluster that can be overlapped. For example, with two triclusters $T_1$ and $T_2$, whose dimensions are $2 \times 2 \times 2$ and $3 \times 3 \times 3$, if $O_{elements} = 50\%$ then the amount of shared elements between the two triclusters will be $50\%$ of the smallest one, $T_1$, so that both will share $0.5 \times 8 = 4$ elements;
5. **Allowed amount of overlapping across rows, columns, or contexts:** the user can also restrict how much of each dimension, relative to the other tricluster, can be used in the overlapping region. By default, these three parameters, $O_{rows}$, $O_{columns}$, and $O_{contexts}$ are set to $100\%$. This way, when two triclusters, $T_1$ and $T_2$, overlap, both can share all observations, all attributes, or all contexts, as long as they respect the restrictions above. On the other end, if $O_{rows} = 50\%$, then, $T_1$ and $T_2$ can still share all attributes and all contexts, but can only share, at maximum, half of their observations.

### Quality

One aspect to take in account in any kind of dataset is its quality. Three-way data is not an exception, as a set of diverse factors can influence how well the dataset can represent reality. These factors can appear in the form of *missing values*, where the dataset does not possess all the elements it should; in *noisy data*, where some values show a slight deviation from what should be the true values; or *errors*, where some elements show unexpected values, huge deviations from expectations or are outliers. These factors can also influence the coherency of a tricluster, since they are not immune to them. In this scenario, some state-of-the-art triclustering algorithms can deal with these factors, mostly with *missings* or *noise* when searching and evaluating a solution. Since this is an important feature, G-Tric allows the user to control the quality of the produced dataset, either on the background data or on the planted triclusters, by adjusting the following parameters:

1. **Percentage of missing values on background:** lets the user define the exact amount of elements on the background, that is, elements that do not belong to any tricluster, that are *missing*. Assuming a dataset *D* with $|D| = 10 \times 10 \times 10 = 1000$ elements, a set triclusters $T = \{t_0, t_1, \ldots , t_n\}$, where $|T| = \sum _{t = 0}^{n} |T_t| = 100$, then the background size will be $|B| = |D| - |T| = 900$. If $M_{background} = 50\%$, then $900 \times 50\% = 450$ values on the background will be replaced by *missing* values;
2. **Percentage of missing values on planted triclusters:** controls the amount of missing values allowed on each triclusters. Unlike $M_{background}$, to facilitate the generation of dynamic structures $M_{triclusters}$ defines only the maximum percentage of elements that can be replaced by *missing* values. For example, given a tricluster *t* with a dimension of $|t| = 2 \times 2 \times 2 = 8$ and $M_{triclusters} = 50\%$, the tricluster will have a number of *missing* values ranging, randomly, between [0, 4];
3. **Percentage of noise on background:** Controls the exact amount of *noise* on the dataset’s background. A given value is considered *noisy* if the difference between it and the original value is smaller than a defined threshold, that is, has to respect the following restriction $|originalValue-noisyValue| \le N_{deviation}$, where $N_{deviation}$ represents the said threshold, that controls how much a value can diverge from the original. More on this parameter below. Using the same dataset *D* defined above, if $N_{background} = 30\%$, then $900 \times 30\% = 270$ values on the background will be replaced by *noisy* values;
4. **Percentage of noise on planted triclusters:** Defines the maximum amount of *noise* that can be present on a tricluster’s values. For the tricluster *t* defined above, if $N_{triclusters} = 25\%$, the tricluster will have a number of *noisy* values ranging, randomly, between [0, 2];
5. **Noise deviation:** Defines the boundary from which *noise* and *errors* can be distinguished. This value can be an *integer*, for *integer* or *symbolic* datasets, or *real-valued* for *non-integer* data. Assuming a *symbolic* dataset *D*, with an alphabet with 10 symbols, $A_D = \{0, 2, 3, \ldots , 9\}$, if $N_{deviation} = 2$ and the current value in study $v = 5$, then, for *v* to become a *noisy* value, it should range between the values which are at a maximum distance of two, in this case, $\{3,4,6,7\}$. If it is replaced by a value at a higher distance, like one of the following, $\{1,2,8,9\}$, then the new value will be considered an error. For both aspects, the new value is chosen randomly from the set of possible ones;
6. **Percentage of errors on background:** Defines the exact amount of *errors* on the background data. In this case, each *error* value is either the minimum of the maximum value or the dataset, considering their distance to the expected value of the selected background distribution;
7. **Percentage of errors on planted triclusters:** Sets the maximum amount of *errors* than can be planted on a triclusters. In this parameter, the $N_{deviation}$ argument is used to generated the new *error* value. On the example dataset *D*, if $E_{background} = 10\%$, then $900 \times 10\% = 90$ values on the background will be considered *errors*.

The definition of the quality level on background data and tricluster data was divided to allow a higher degree of customization as well as to encourage the generation of flexible structures with varying properties.

### Output

After the generation is completed, the generator outputs three files: the first one, with the suffix *“_data“*, contains the dataset in a tab-separated format. The last ones, with the suffix *“_trics“* and in txt and JSON formats, contains information about the number of triclusters planted, including their number, their *coverage* (how many elements in the dataset belong to the triclusters), and for each one indicates its size, its location, by showing the corresponding set of rows, columns, and contexts, the pattern associated to each dimension and the percentage of missing, noise and errors planted in it. The file also shows the percentage of missing values, noise, and error on the whole dataset. The user can also visualize the output using a G-Tric’s feature that uses a graphical tool to generate heatmaps representing the tricluster’s slices.

## Results

As described in the previous section, G-Tric makes available a wide set of custom features allowing the creation of several types of datasets, diverging either in their nature, structure, or on how coherent their hidden subspaces are. These set of features allow also the user to simulate the properties of existing datasets.

Together with the generator, this work provides, two sets of publicly available datasets: the first contains a simulation of real world data, replicating the characteristics of the datasets listed in Table 2, so that further comparisons made with that data can have the plus of considering the ground truth; while the second set contains a group of datasets designed to test and evaluate some behavioral specificities of triclustering algorithms.

**Table 2:** Example datasets used during the development of some of the existing algorithms

	Dataset type	Description	Dimensions
1	Gene expression	Yeast cell cycle (elucitration) [39]	7679 $\times$ 13 $\times$ 14
2	Financial	Stock market ratios [40]	3200 $\times$ 30 $\times$ 28
3	fMRI	Average blood-oxygen-level-dependent contrast [41]	20 $\times$ 464 $\times$ 94
4	Social	Bibsonomy [42]	51 $\times$ 924 $\times$ 2844
5	Georeferenced time-series	Dutch daily average temperature [43]	28 $\times$ 20 $\times$ 365

Table 2 describes a set of sample datasets used by triclustering authors when developing and testing their algorithms [2, 6, 7, 35, 36]. These datasets show the range of possibilities covered by G-Tric, since they describe different domains, from genetics, to health records without forgetting social, economic, or geographic use cases. They show different data types, from binary data, describing the existence of triples, to real-valued data reflecting expression values. They further present distinct patterns present on the existing subspaces, such as the ones presented in section "Background", as well as distinctive levels of quality. Finally, each dataset explores a different dimension. Biological datasets, for example, generally have a higher number of genes (observations) than samples (features), while in health records, the number of patients (observations) is usually a lot smaller than the number of features.

Dataset 1 was collected from the yeast cell cycle data for the Elutritration experiments and describes a set of genes whose expression value is measured from time 0 to 390 min at 30-min intervals (14-time points) across 13 attributes. Dataset 2 summarizes 28 years of financial figures and price data from all North America that were converted to 30 financial ratios for each of the 3200 stocks. Dataset 3 collected brain fMRI data from 20 male subjects at rest over 94-time points. The blood flow (BOLD) intensity was measured at every voxel of the brain along time, providing levels of some 100,000 voxels every 2–3 s, where standard fMRI preprocessing was applied. Dataset 4 shows a random sample of 3000 of the first 100,000 triples of the bibsonomy.org dataset, where objects are users, attributes are tags, and conditions are bookmark names. Each triple contributes to a system developed for sharing bookmarks and publications easily. Dataset 5 combines temperature data collected from 28 dutch meteorological stations each day, over 20 years.

These examples meant to demonstrate the set of properties that can be shaped by G-Tric, allowing the simulation of exiting data with two main advantages: the first is that even when simulating existing data, the fact of possessing the ground truth allows extracting more from the comparisons done with this data. There is no longer the limitation of being restricted to the intrinsic quality of the solutions. Intrinsic quality can be further combined with the extrinsic evaluation of data that expresses the same characteristics as the real one and has more information than the simple synthetic datasets that were currently used.

The second advantage is the possibility to explore how the algorithms perform on specific tasks. After performing a general evaluation of each solution’s quality, the authors can now effortlessly test or evaluate their algorithms under specific circumstances. For example, to assess how their methods respond on scenarios where one or all dimensions are large, have low quality (high number of missings and the presence of noise and errors), show high levels of overlapping between subspaces, or to simply test its ability to catch some specific type of patterns. This is useful when developing an algorithm whose goal is to improve an existing approach in some aspects.

### Simulating real data

For each dataset, some settings were collected from the data, such as, dimensions, data type, size of the alphabet and the amount of missing values. However, since there is no ground truth available, most of the settings regarding triclusters, overlapping, and quality properties are unknown. We complemented the characterization of triclustering properties provided in the survey by Henriques and Madeira [1] with the conclusions drawn by different authors, [2, 5–7, 35, 37, 38], when applying their triclustering algorithms to the chosen datasets, summarized in Table 3 and detailed below.

**Table 3:** Settings to simulate the real datasets

Properties	Dataset 1	Dataset 2	Dataset 3	Dataset 4	Dataset 5
Dataset					
Data type	Real valued	Real valued	Real valued	Binary	Real valued
Dimensions	7679 × 13 × 14	3200 × 30 × 28	20 × 494 × 94	51 × 924 × 2844	28 × 20 × 365
Alphabet	[0, 500]	[5, 1000]	[−5, 5]	0, 1	[−10, 30]
Background	Uniform	Norma l(500, 150)	Uniform	Discrete (0.7, 0.3)	Normal (14, 7)
Missings	0%	20%	0%	0%	15%
Noise	0%	10%	20%	0%	20%
Errors	0%	10%	15%	0%	5%
Triclusters					
Number	7	10	5	7000	128
Dimensions	U(80, 400) × U(2, 4) × U(3, 13)	U(100, 500) × U(10, 20) × U(5, 15)	U(5, 15) × U(50, 200) × U(15, 50)	U(5, 8) × U(20, 70) × U(100, 400)	U(4, 4) × U(4, 4) × U(8, 8)
Contiguity	No	No	No	No	No
Patterns	All types	All types	All types	Constant	All types
Missings	0%	10%	0%	0%	5%
Noise	0%	15%	10%	0%	10%
Errors	0%	5%	5%	0%	2%
Noise deviation	0	2	1	0	1
Overlapping					
Plaid coherency	No overlapping	Additive	Additive	None	No overlapping
% Overlapping trics	0%	40%	100%	80%	0%
Max. interactions	0	2	3	300	0
% Overlapping elems.	0%	50%	40%	70%	0%
Restrictions on rows/columns/contexts	0%/0%/0%	100%/100%/100%	100%/100%/100%	100%/80%/80%	0%/0%/0%

Dataset 1 was used by the following algorithms: *Tricluster* [2], *THD-Tricluster* [5], *SubCubeMiner* [37], and *SS-Sim-Tri* [38]. When analysing the respective results we noticed that the number of triclusters vary between 4 and 6, with a clear dominance of the gene dimension without any overlapping reported. Although these results differ from algorithm to algorithm and there is no ground truth to validate them, they are useful for approximating the data’s real characteristics.

Dataset 2 was used by *CATSeeker* [7] and reported 20% of missing values across the dataset, without explaining the number of found triclusters, since the analysis was only focused on the average return value of the clustered stocks.

Dataset 3 was used by *TWIGS* [35] and reported five core modules (triclusters) with overlapping subjects and parcels between them.

Dataset 4 was tested using both *OAC-Triclustering (box and primes)* [6] algorithm and reported an average of 7000 triclusters with overlapping regions across the 3000 triples available on the dataset.

Finally, Dataset 5 has a real-valued alphabet, representing the temperature values, restricted between − 10 and 30, and reported 128 regular triclusters with the same dimensions ($4 \times 4 \times 8$). The remaining settings were all filled with values that, although they cannot guarantee the dataset’s identity, try to make them as interesting as the original ones.

### Testing algorithm’s properties

A first group of *clean* datasets was created without any noise or overlapping. These are supposed to serve as *base* datasets for the next ones that will be created to test each property, and consist of four datasets, one for each data type (R-Real-valued, S-Symbolic, B-Binary and C-Integer and Contiguous). Table 4 summarizes the structure of these datasets.

**Table 4:** Settings for the base datasets

Properties	Dataset R	Dataset S	Dataset B	Dataset C
Dataset				
Data type	Real valued	Symbolic	Binary	Integer
Dimensions	1000 × 100 × 10	1000 × 100 × 10	1000 × 100 × 10	1000 × 100 × 10
Alphabet	[−100, 100]	{1, 2, 3, 4, 5}	{0, 1}	[0, 100]
Background	Normal (0, 30)	Discrete (0.1, 0.15, 0.3, 0.3, 0.15)	Uniform	Uniform
Missings	0%	0%	0%	0%
Noise	0%	0%	0%	0%
Errors	0%	0%	0%	0%
Triclusters				
Number	12	12	12	12
Dimensions	U(30, 50) × U(5, 10) × U(2, 4)	U(30, 50) × U(5, 10) × U(2, 4)	U(30, 50) × U(5, 10) × U(2, 4)	U(30, 50) × U(5, 10) × U(2, 4)
Contiguity	No	No	No	On contexts
Patterns	All types	Order preserving, constant	Contant	All types
Missings	0%	0%	0%	0%
Noise	0%	0%	0%	0%
Errors	0%	0%	0%	0%
Noise deviation	0	0	0	0
Overlapping				
Plaid coherency	No overlapping	No overlapping	No overlapping	No overlapping
% Overlapping trics	0%	0%	0%	0%
Max. interactions	0	0	0	0
% Overlapping elems.	0%	0%	0%	0%
Restrictions on rows/columns/contexts	0%/0%/0%	0%/0%/0%	0%/0%/0%	0%/0%/0%

The first property to test is scalability, that is, how the algorithm handles different data sizes, through its execution time, or memory consumption. The base datasets, of dimension $1000\times 100\times 10$ have $10^6$ elements. Three additional datasets *S*1, *S*2 and *S*3 were created for each type, and containing patterns of all types, with, respectively, $10^7$, $10^8$ and $10^9$ elements. The number of triclusters increases linearly with the dataset’s size. Table 5 summarizes these settings.

**Table 5:** Different dataset sizes to test scalability

Dataset	Base	S1	S2	S3
Dimensions	1000 × 100 × 10	1000 × 100 × 100	10,000 × 100 × 100	10,000 × 1000 × 100
Num. of elements	10^6^	10^7^	10^8^	10^9^
Num. of triclusters	12	120	1200	12,000

The next property to test is the ability to find different types of patterns. To this aim, another set of datasets was generated for each of the four types. At each set, there is one dataset per pattern type, that is, one dataset with only additive patterns, another with only multiplicative patterns, and so on. For Symbolic datasets, the only set of patterns available are the *Order Preserving* and the *Constant* ones. For Binary, only the *Constant* type was used.

**Table 6:** Settings for datasets with low and high level of overlapping

Overlapping	Low	High
% Overlapping trics	30%	75%
Max. interactions	2	3
% Overlapping elems.	30%	70%
Restrictions (|*X*|/|*Y*|/|*Z*|)	100%/70%/70%	100%/100%/100%
Plaid coherency	R: additive, S: none, B: none, C: multiplicative	

Another interesting task is to assess how well algorithms find individual triclusters on an overlapped subspace, with a varying number of interactions between triclusters. In this context, another group of datasets was created, based on the *base* datasets, but with two degrees of overlapping: a low one, where only 30% of the triclusters can overlap (4 of the total 12) and can only interact with another one sharing no more than 30% of their elements. And a high one with 75% of overlapping (9 triclusters of 12), where each one can now overlap other two and share up to 80% of its values. This turns the planted triclusters very similar, making harder for the algorithm to find them. Table 6 summarizes these settings.

The last property to test is how they cope with low-quality data, where the number of missings and noise/errors can influence a subspace’s coherency degree. Similar to what as made with the overlapping property, a new set of datasets was generated with two levels of quality. These settings are also summarized in Table 7.

**Table 7:** Quality settings for datasets with low and high levels of missings, noise and errors

Quality	Higher				Lower			
Dataset type	R	S	B	C	R	S	B	C
Dataset								
Missings	2%	2%	2%	2%	10%	10%	10%	10%
Noise	10%	10%	10%	10%	30%	30%	30%	30%
Errors	5%	5%	0%	5%	15%	15%	0%	15%
Triclusters								
Missings	2%	2%	2%	2%	5%	5%	5%	5%
Noise	10%	10%	10%	10%	20%	20%	20%	20%
Errors	5%	5%	0%	5%	8%	8%	0%	8%
Noise deviation								
Real-valued	1	1	1	1	3	1	1	3

**Table 8:** Rich dataset’s settings

Properties	Dataset R	Dataset S	Dataset B	Dataset C
Dataset				
Data type	Real valued	Symbolic	Binary	Integer
Dimensions	1000 × 100 × 100	1000 × 100 × 100	1000 × 100 × 100	1000 × 100 × 100
Alphabet	[−100, 100]	{1, 2, 3, 4, 5}	{0, 1}	[0, 100]
Background	Normal (0, 30)	Discrete (0.1, 0.15, 0.3, 0.3, 0.15)	Uniform	Uniform
Missings	2%	2%	2%	2%
Noise	10%	10%	10%	10%
Errors	5%	5%	0%	5%
Triclusters				
Number	30	30	30	30
Dimensions	U(30, 50) × U(5, 10) × U(3, 5)	U(30, 50) × U(5, 10) × U(3, 5)	U(30, 50) × U(5, 10) × U(3, 5)	U(30, 50) × U(5, 10) × U(3, 5)
Contiguity	No	No	No	On contexts
Patterns	All types	Order preserving, constant	Contant	All types
Missings	2%	2%	2%	2%
Noise	10%	10%	10%	10%
Errors	5%	5%	0%	5%
Noise deviation	2	1	1	2
Overlapping				
Plaid coherency	Additive	None	None	Multiplicative
% Overlapping trics	50%	40%	60%	60%
Max. interactions	3	2	3	4
% Overlapping elems.	60%	50%	80%	70%
Restrictions on rows/columns/contexts	100%/100%/100%	100%/100%/100%	100%/100%/100%	100%/100%/100%

A last group of datasets were generated, now combining all the properties tested so far, making them richer when compared with the *base* ones. This set of *rich* datasets are described in Table 8.

The datasets are available at the project repository (see section "Availability and requirements") inside a demo project that serves as an example of a programmatic way to use G-Tric. Additional file 1 provides an example of a use case for G-Tric, by simulating the generation of one of the presented datasets.

## Conclusions

Triclustering has recently gained increasing importance as an effective supervised learning approach to three-way data analysis. In this context, where the number of triclustering algorithms exploded in recent years, and each algorithm tries to improve the state-of-the-art generating their own datasets, comparisons and benchmarks get even more crucial.

G-Tric is capable of generating three-way with embedded triclustering solutions, with a varied range of properties that can be controlled, and patterns that can be explored. Its goal is to help surpassing the limitations of current evaluation using real world data, promoting the quality of research in triclustering and improving the quality of results.

The primary way to perform such evaluation is through the analysis of the method’s performance on data. Although abundant, real-world data is quite limited on the type of inherent properties and on the kind of conclusions that can be drawn from an execution. Since no ground truth is usually available for real datasets, and different methods are designed to find different solutions, these solutions can only be evaluated through their intrinsic quality, not against one another. This limits the number and quality of conclusions that can be drawn when evaluating new triclustering approaches.

The generation of synthetic data helps to overcome this problem. However, existing generated data can show biases towards specific triclustering solutions, making them bad benchmarks for other solutions. Furthermore, these datasets are often not made available to the scientific community.

In this context, we propose G-Tric, a synthetic three-way data generator allowing the creation of synthetic datasets with configurable properties and the possibility to plant triclusters. The generator is prepared to create datasets resembling real 3-way data from biomedical and social data domains, with the additional advantage of possessing the ground truth.

To our knowledge, G-Tric is the first contribution of this kind for 3D data, it was inspired by existing contributions for biclustering, improving many of their drawbacks. Together with G-Tric, we make available a set of datasets, with predefined settings, mimicking specific types of real-world data, supporting the development and testing of new triclustering algorithms by enabling the community to accurately compare their methods’ solutions with triclustering solutions provided as grouth truth with the generated three-way datasets.

Besides the ability to easily generate customized three-way data with triclustering solutions, the proposed generator enables the possibility to perform benchmarks on existing algorithms to study their efficiency within certain conditions, or their effectiveness in finding different types of patterns, by allowing the creation of several datasets with an extensive board of characteristics. This provides the unprecedented opportunity to comprehensively assess the strengths and limitations of state-of-the art and new triclustering algorithms, promoting the advance in the area of three-way data analysis.

## Availability and requirements

**Project name:** G-Tric

**Project home page:** https://github.com/jplobo1313/G-Tric

**Operating system(s):** Platform Independent

**Programming language:** Java

**Other requirements:** Java 11 or above

**License:** GNU GPLv3

**Any restrictions to use by non-academics:** No

## Supplementary information

**Additional file 1.** Dataset generation using G-Tric. Guide exemplifying G-Tric’s interface and demonstrating, step by step, how datasets can be generated.

## Data Availability

The datasets generated during and/or analysed during the current study are available in the G-Tric repository, https://github.com/jplobo1313/G-Tric.

## Footnotes

1 https://github.com/zakimjz/IBMGenerator.

2 https://github.com/knowm/XChart.
