# Supplementary material for: G-Tric: generating three-way synthetic datasets with triclustering solutions
Source: BMC Bioinformatics. 2021 Jan 7;22:16. doi: 10.1186/s12859-020-03925-4 (PMC7789692; doi:10.1186/s12859-020-03925-4)
Supplement: Supplementary file 1 — Additional file 1. Dataset generation using G-Tric. Guide exemplifying G-Tric’s interface and demonstrating, step by step, how datasets can be generated. [file 12859_2020_3925_MOESM_ESM.pdf]

Additional File 1: Dataset generation using G-Tric

This file introduces the use of G-Tric's *user interface* by presenting a use case, where the symbolic dataset, S, in the main manuscript's Table 8 is generated step by step, using the settings described in the implementation section. Each of the following sections shows the different stages of the generator. In the end, the output produced is analyzed.

Dataset Properties

The first step is to define the set of properties that will characterize dataset S. This can be done through the *"Dataset Properties"* tab on the interface, as exemplified in Figure 1. Dataset S will be composed by 1000 observations, 100 attributes and 100 contexts, thus the parameters *Number of Rows* (1), *Number of Columns* (2) and *Number of Contexts* (3) have the same values.

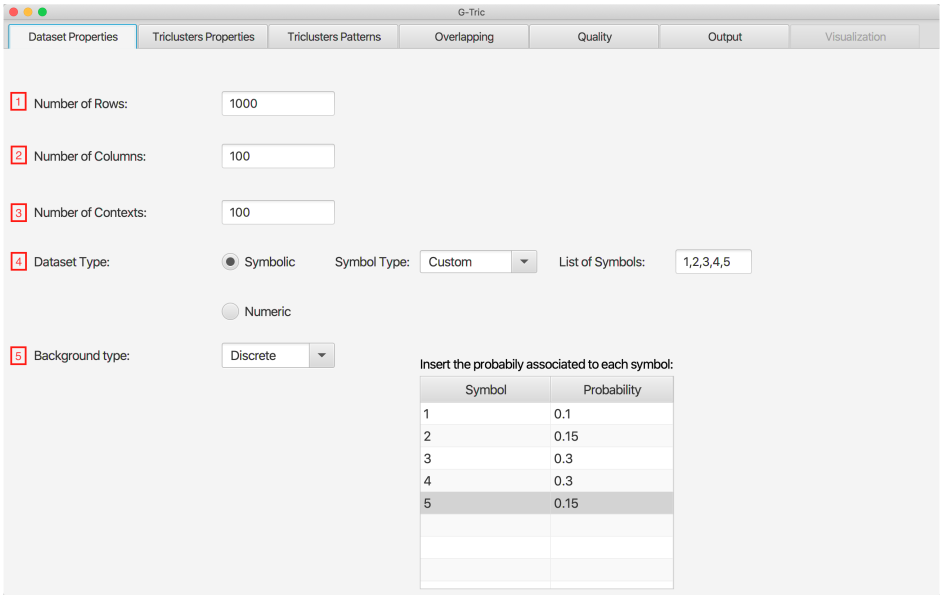

Figure 1 G-Tric: Dataset properties tab.

The parameter *Dataset Type* (4) then sets the type of values that constitute the dataset, that can be either *symbolic* or *numeric*. If the first one is chosen, the user will have to indicate if the alphabet is composed of default symbols, generated automatically, where the user only indicates the alphabet size. Alternatively, if he/she desires a custom alphabet, the list of symbols will be required. In this case, the user selects {1,2,3,4,5} as the target alphabet. The order of the symbols in the input determines the ordering of the alphabet, that in this case will be $1 < 2 < 3 < 4 < 5$. On the latter, the user can define if the numeric alphabet is represented by either real-valued or integer values, and defines the allowed range of values.

The last parameter, *Background* (5), allows the user to choose between four possible types to determine how the background values of the dataset are distributed: *Uniform*, *Normal*, *Discrete* and *Missing*. If the user chooses *Normal*, or *Discrete*, additional parameters are presented to set the distributions parameters, like the

Mean and *Standard Deviation* on the *Normal* option, or a table with an editable probability is associated to each symbol, for the *Discrete* one. As described by Figure 1. Dataset S will have a discrete background with the following probabilities: {1: 0.1, 2: 0.15, 3: 0.3, 4: 0.3, 5: 0.15}.

Tricluster Properties

The next step defines the amount and the structure of the planted triclusters on the dataset to be generated. The number of triclusters in dataset S can be defined through parameter *Number of triclusters* (1).

The following three sets of parameters define their structure: Row (1)/Column (2)/Context (3) distribution and respective parameters. The user has available two types of distributions: *Normal* and *Uniform*. The interface dynamically adapts the respective parameters to ask for *Mean* and *Standard Deviation* for the first type, and *Min* and *Max* for the second one. For dataset S, its structure follows a uniform distribution, and each tricluster will have a set of rows, columns, and contexts varying between $[30, 50]$, $[5, 10]$ and $[3, 5]$, respectively.

The last parameter, *Contiguity* (5), enables the selection on whether the planted triclusters should be contiguous across the column or context dimension. In this case, dataset S's triclusters will will not be contiguous along these dimensions.

Figure 2 exemplifies the tricluster's properties tab.

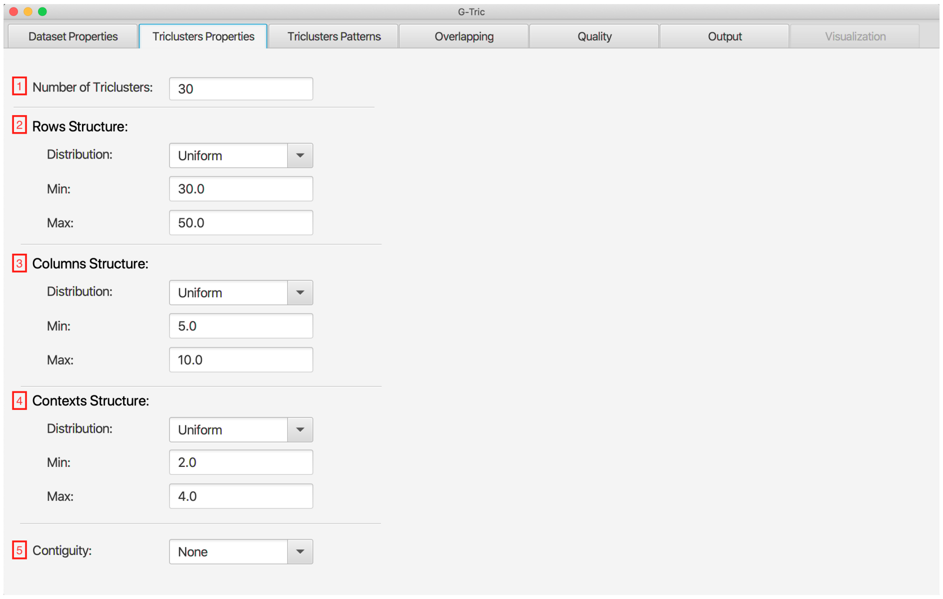

Figure 2 G-Tric: Tricluster properties tab.

Tricluster Patterns

We now focus the set of patterns that will be expressed by the set of triclusters planted. The number of patterns chosen will be uniformly distributed across the set of tricluster available. For example, if the user sets four patterns, and the dataset has eight triclusters, two biclusters will be assigned to each type.

Dataset S will have every existing pattern following the *Order Preserving* and *Constant* types, as presented on the background section. As for the *Order Preserving* pattern on contexts, the user is able to select whether the generated temporal pattern can have an arbitrarily number of increases and decreases along time, or follow a monotonically increasing or decreasing pattern. The GUI makes available an example image, as in Figure 3, for each pattern, to described it and help the user choosing what he/she desired. In this case study, this option is set to *Random*. Figure 4 exemplifies the tricluster's pattern tab.

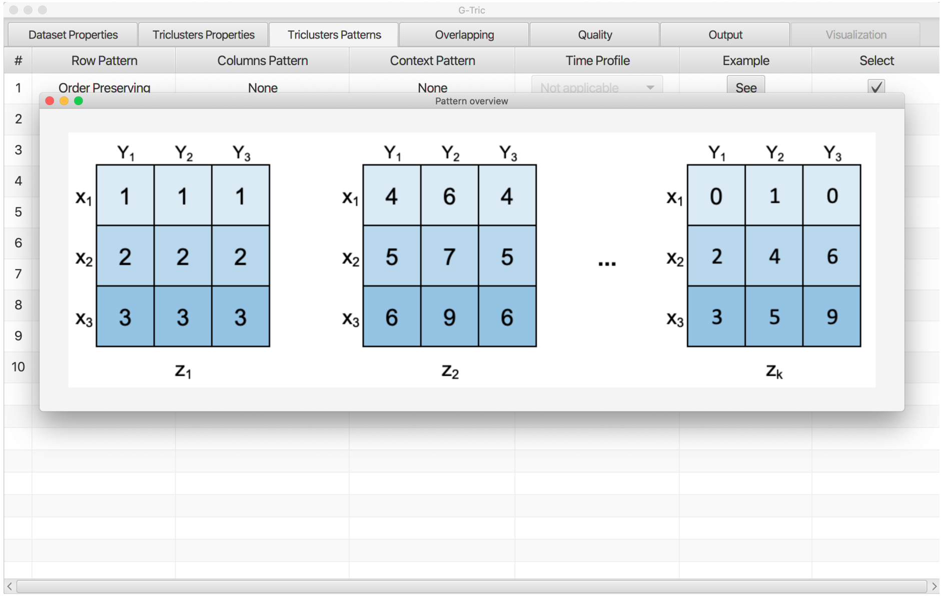

Figure 3 G-Tric: Illustrative example of a pattern.

Overlapping

The *Overlapping* tab, shown in Figure 5, allows the user to define the number of triclusters that are allowed to overlap and how their interactions are expressed. This interaction is controled by the first parameter *Plaid Coherency* (1), that makes available the five types presented earlier: *Additive*, *Multiplicative*, *Interpoled*, *None* and *No Overlapping*. For dataset S the *None* plaid coherency will be chosen.

The second step is to set the amount planted triclusters that can overlap. This is done through parameter *% of Overlapping Triclusters* (2). For dataset S, only 12 of the 30 planted triclusters can overlap, so this parameter will be set to 40%.

Then the user has to define how the overlapped triclusters will interact with each other. This is done, first, by defining the maximum number of subspaces that can overlap simultaneously, using the parameter *Maximum Number of Triclustering Interactions* (3). Then the user defines how many elements two overlapped triclusters can share, using parameter *% of Overlapping Elements Between Triclusters* (4). Each tricluster on dataset S can overlap with another one, so the number of simultaneous interactions is 2. A set of triclusters can also share 50% of the smallest tricluster's elements. The last three parameters allow the introduction of restrictions on the number of rows (5), columns (6), and contexts (7) that can be shared

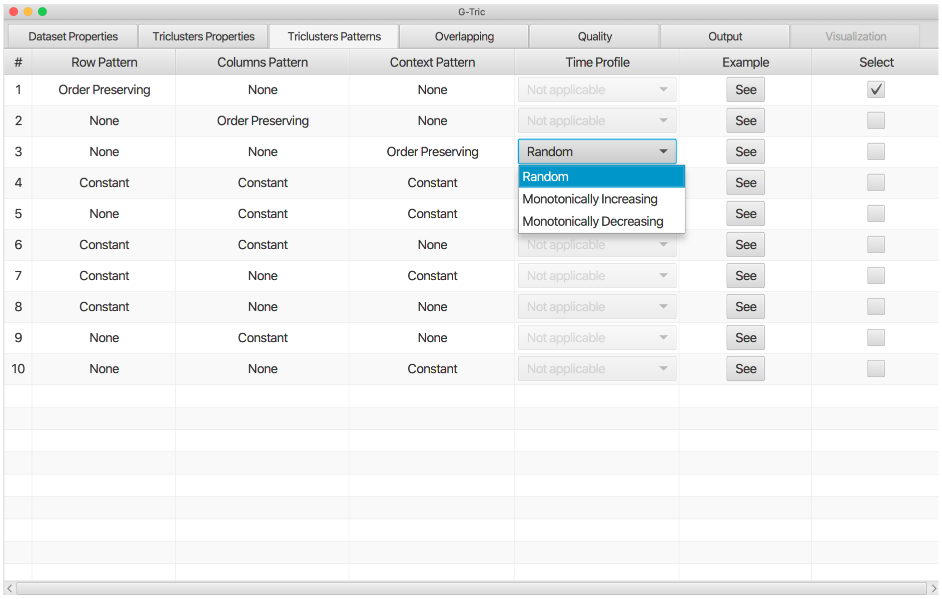

Figure 4 G-Tric: Tricluster patterns tab.

by a set of overlapping triclusters. Since dataset S has triclusters with smaller attribute and context dimensions, we decided not to apply any restriction, so all three parameters were set to 100%.

Quality

The *Quality* tab, illustrated in Figure 6, controls properties from the dataset and the triclusters. Here the user can define the amount of missing values, noise, and errors on both dataset's background and planted triclusters.

For dataset S, the *% of Missing Values on Background* (1) is set to 2% percent, while the *% of Missing Values on Planted Triclusters* (2) is also 2%. This means that each tricluster will have, at maximum, 2% of its elements missing. For noise, the *% of Noise on Background* (3) and the *% of Noise on Planted Triclusters* (4) is 10%. Here, parameter (4) controls the maximum amount of noisy elements, just as above. The *Noise Deviation* (5) is set to 1. This means that the noisy value will be, at maximum, at a distance of 1 from the original value. The last setting defines the proportion of errors on the dataset. The *% of Errors on Background* (6) and the *% of Errors on Planted Triclusters* is set to 5%. The error elements will be at a distance from the original values of at least the value of *Noise Deviation* (5).Parameters (1), (3), and (6) control the exact amount of missing values, noise, and errors in the background.

Output

The last stage before generating the new dataset is defining how and where the output will be stored, as resumed in Figure 7. The first parameter, *Save On* (1) allows the user to decide whether the dataset should be stored on a single or onyo multiple files. Multiple files are worth it when the dataset has large dimensions, since it can be divided in small chunks across several files. The second parameter,

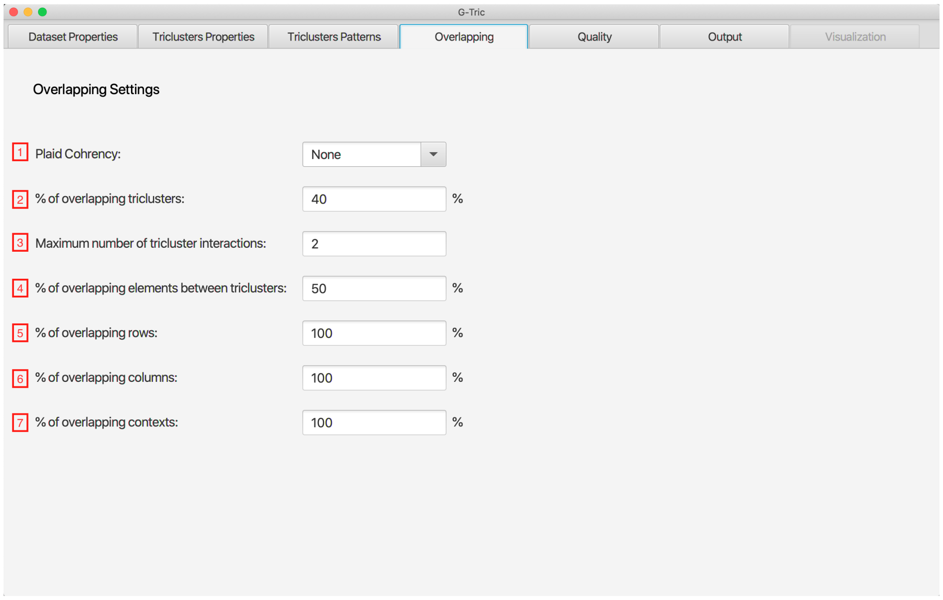

Figure 5 G-Tric: Overlapping settings tab.

File Name (2), sets the prefix of the name of all three output files. The first file will contain the dataset in a *tsv* format, with the values separated by a tab delimiter, as shown in Figure 8. The remaining two files will contain the information about the triclusters planted on either *txt* format, illustrated in Figure 9, where some statistics and the summary of the first tricluster, as well as the content for the first context is shown; and also by a *JSON* format, as shown in Figure 10. The last parameter, *Save to Directory* (3), specifies where the output will be stored.

Visualization

The last tab of the application allows the user to visualize the output, by showing the triclusters that resulted from the generation process. Figure 11 shows the visualization options. This tab is composed by two sections: 1) One with the information regarding the tricluster's structure, and 2) one with a graphical representation of each tricluster's slice.

As the user chooses one of the available triclusters (1), the left section of the interface (2) shows information that describes the planted subspace, such as, its dimensions, where it is located (on which rows, columns and contexts), which are the patterns followed by each dimension, and respective factors, when available (only in additive or multiplicative patterns), the plaid coherency assumed and the degree of missing values, noise and errors.

The right section (3) displays a table with each one of the tricluster's slices, that is, the contexts where it is present. The user can visualize the values of each context through a new windows that displays a graphical representation of the slice using a heatmap, that easily reflects the pattern expressed, as shown in Figure 12. In this case, the figure presents the visualization of the first context (No. 47) of the tricluster with an *Order Preserving* pattern on rows. This can be confirmed by order-preserving patterning of colors for each row across columns

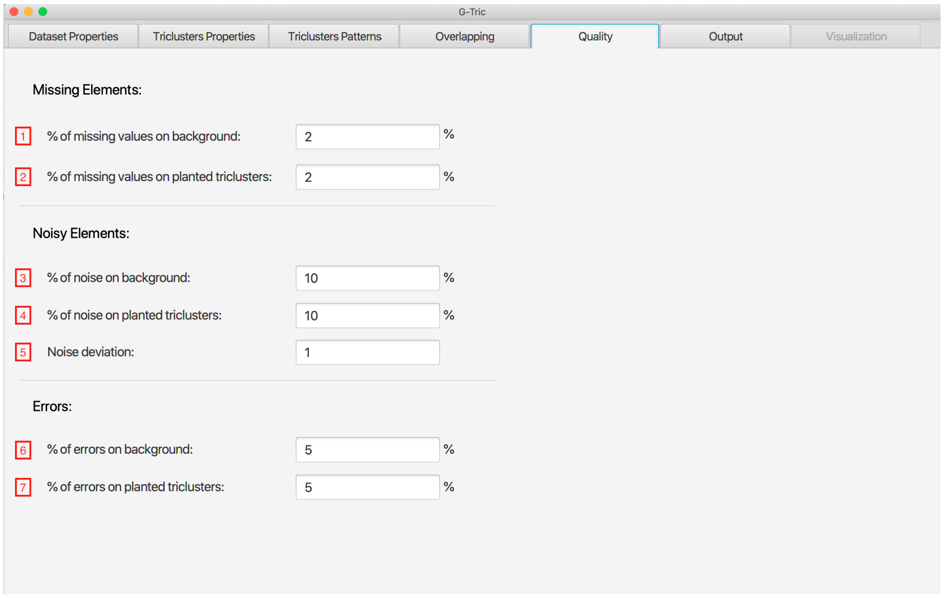

Figure 6 G-Tric: Quality settings tab.

$J = \{y_1, y_{15}, y_{24}, y_{33}, y_{42}, y_{53}, y_{95}\}$. This particular slice presents only one missing value, represented in white, as well as other deviations from the expected values caused by noise and errors.

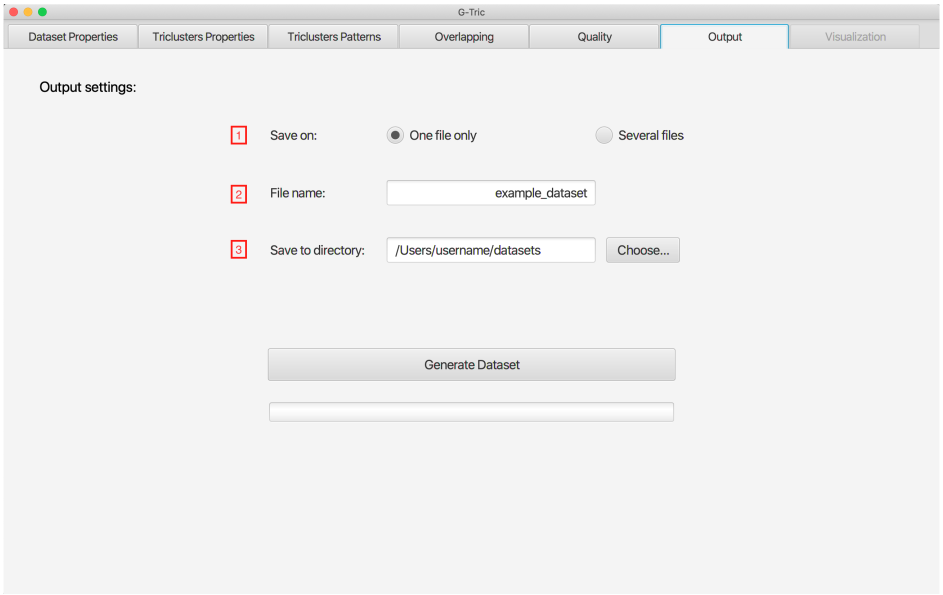

Figure 7 G-Tric: Output tab.

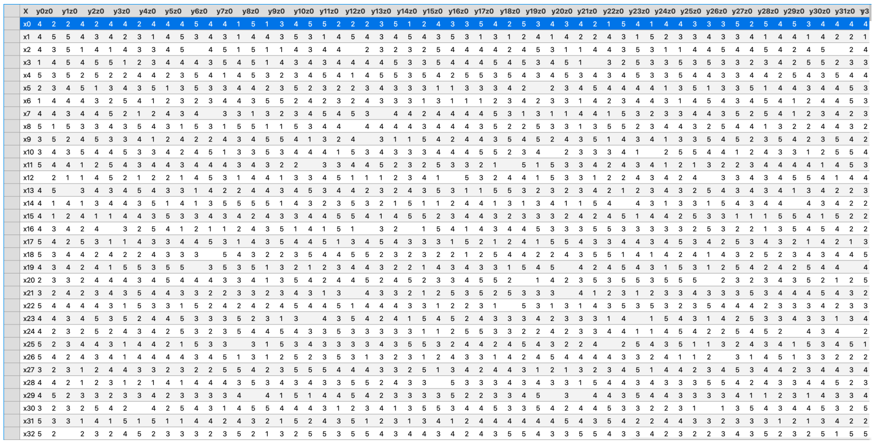

Figure 8 G-Tric: Dataset tsv file.

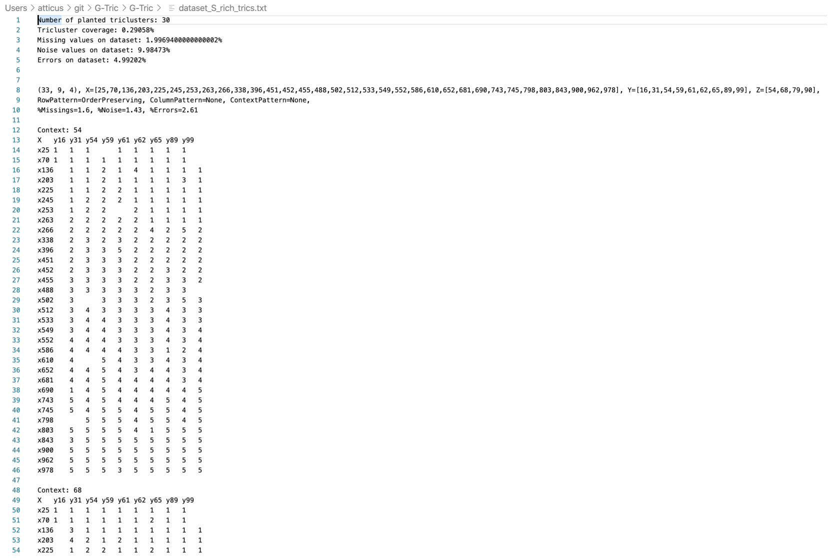

Users > atticus > git > G-Tric > G-Tric > dataset_S_rich_trics.txt

```
Number of planted triclusters: 30
Tricluster coverage: 0.29058%
Missing values on dataset: 1.9969400000000002%
Noise values on dataset: 9.98473%
Errors on dataset: 4.99202%


(33, 9, 4), X=[25,70,136,203,225,245,253,263,266,338,396,451,452,455,488,502,512,533,549,552,586,610,652,681,690,743,745,798,803,843,900,962,978], Y=[16,31,54,59,61,62,65,89,99], Z=[54,68,79,90],
RowPattern=OrderPreserving, ColumnPattern=None, ContextPattern=None,
%Missings=1.6, %Noise=1.43, %Errors=2.61

Context: 54
X   y16 y31 y54 y59 y61 y62 y65 y89 y99
x25 1   1   1       1   1   1   1   1
x70 1   1   1   1   1   1   1   1   1
x136    1   1   2   1   4   1   1   1   1
x203    1   1   2   1   1   1   1   3   1
x225    1   1   2   2   1   1   1   1   1
x245    1   2   2   2   1   1   1   1   1
x253    1   2   2       2   1   1   1   1
x263    2   2   2   2   2   1   1   1   1
x266    2   2   2   2   2   4   2   5   2
x338    2   3   2   3   2   2   2   2   2
x396    2   3   3   5   2   2   2   2   2
x451    2   3   3   3   2   2   2   2   2
x452    2   3   3   3   2   2   3   2   2
x455    3   3   3   3   2   2   3   3   2
x488    3   3   3   3   3   2   3   3
x502    3       3   3   3   2   3   5   3
x512    3   4   3   3   3   3   4   3   3
x533    3   4   4   3   3   3   4   3   3
x549    3   4   4   3   3   3   4   3   4
x552    4   4   4   3   3   3   4   3   4
x586    4   4   4   4   3   3   1   2   4
x610    4       5   4   3   3   4   3   4
x652    4   4   5   4   3   4   4   3   4
x681    4   4   5   4   4   4   4   3   4
x690    1   4   5   4   4   4   4   4   5
x743    5   4   5   4   4   4   5   4   5
x745    5   4   5   5   4   5   5   4   5
x798        5   5   5   4   5   5   4   5
x803    5   5   5   5   4   1   5   5   5
x843    3   5   5   5   5   5   5   5   5
x900    5   5   5   5   5   5   5   5   5
x962    5   5   5   5   5   5   5   5   5
x978    5   5   5   3   5   5   5   5   5

Context: 68
X   y16 y31 y54 y59 y61 y62 y65 y89 y99
x25 1   1   1   1   1   1   1   1   1
x70 1   1   1   1   1   1   2   1   1
x136    3   1   1   1   1   1   1   1   1
x203    4   2   1   2   1   1   1   1   1
x225    1   2   2   1   1   2   1   1   1
```

Figure 9 G-Tric: Tricluster's txt file.

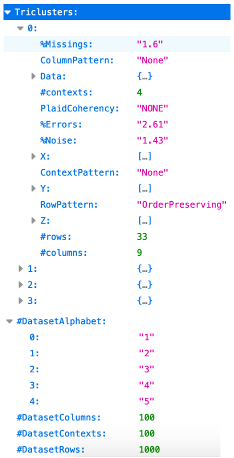

Triclusters:
0:
%Missings: "1.6"
ColumnPattern: "None"
Data: {…}
#contexts: 4
PlaidCoherency: "NONE"
%Errors: "2.61"
%Noise: "1.43"
X: […]
ContextPattern: "None"
Y: […]
RowPattern: "OrderPreserving"
Z: […]
#rows: 33
#columns: 9
1: {…}
2: {…}
3: {…}
#DatasetAlphabet:
0: "1"
1: "2"
2: "3"
3: "4"
4: "5"
#DatasetColumns: 100
#DatasetContexts: 100
#DatasetRows: 1000

Figure 10 G-Tric: Tricluster's JSON file.

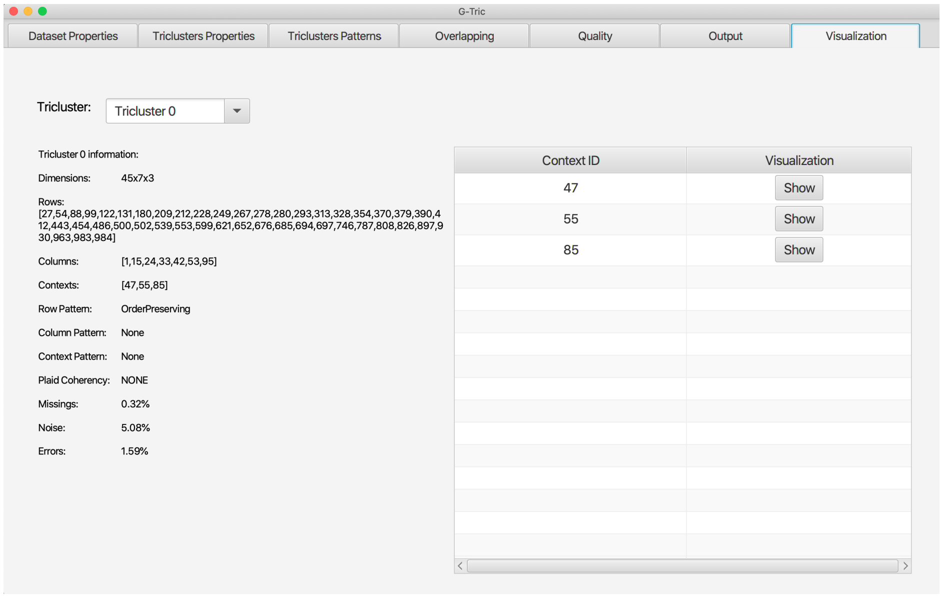

Figure 11 G-Tric: Tricluster's summary

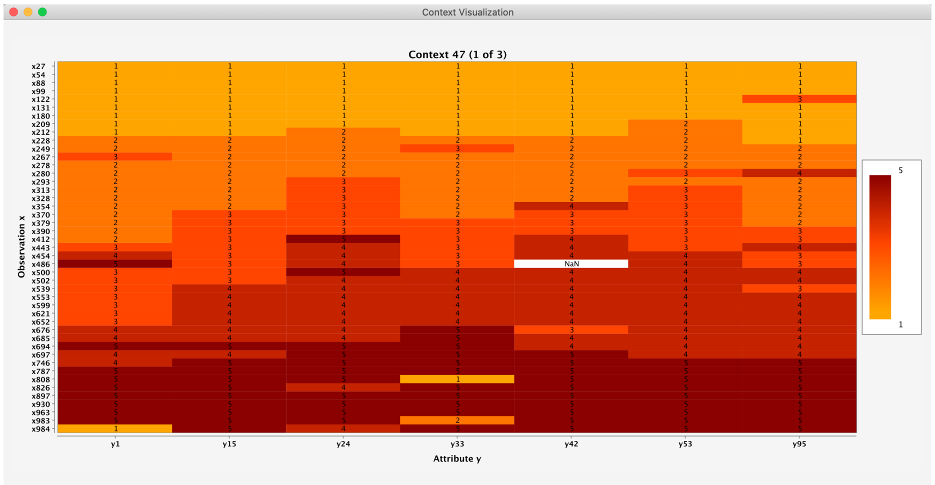

Figure 12 G-Tric: Heatmap to visualize a tricluster's pattern
